# Broad-Spectrum Antiviral Natural Products from the Marine-Derived *Penicillium* sp. IMB17-046

**DOI:** 10.3390/molecules24152821

**Published:** 2019-08-02

**Authors:** Jiao Li, Yujia Wang, Xiaomeng Hao, Shasha Li, Jia Jia, Yan Guan, Zonggen Peng, Hongkai Bi, Chunling Xiao, Shan Cen, Maoluo Gan

**Affiliations:** 1 Institute of Medicinal Biotechnology, Chinese Academy of Medical Sciences and Peking Union Medical College, Beijing 100050, China; 2 Department of Pathogen Biology, Jiangsu Key Laboratory of Pathogen Biology, Nanjing Medical University, Nanjing 210029, China

**Keywords:** marine fungus, *Penicillium*, pyrazine natural product, antiviral, HIV, influenza A virus, *Helicobacter pylori*

## Abstract

A new pyrazine derivative, trypilepyrazinol (**1**), a new α-pyrone polyketide, (+)-neocitreoviridin (**2**), and a new ergostane analogue, 3β-hydroxyergosta-8,14,24(28)-trien-7-one (**3**), were isolated and characterized along with five known compounds from the marine-derived fungus *Penicillium* sp. IMB17-046. The structures of these new compounds were determined using spectroscopic data analyses (HRESIMS, 1D- and 2D-NMR), X-ray crystallography analysis, and TDDFT ECD calculation. Compounds **1** and **3** exhibited broad-spectrum antiviral activities against different types of viruses, including human immunodeficiency virus (HIV), hepatitis C virus (HCV), and influenza A virus (IAV), with IC_50_ values ranging from 0.5 to 7.7 μM. Compounds **1** and **2** showed antibacterial activities against *Helicobacter pylori*, a causative pathogen of various gastric diseases, with minimum inhibitory concentration (MIC) values of 1–16 μg/mL.

## 1. Introduction

Viruses such as the human immunodeficiency virus (HIV), influenza virus, and hepatitis B and C viruses (HBV and HCV) are great global threats to public health. According to the recent World Health Organization (WHO) reports, 36.7 million people were living with HIV in 2015, among which approximately 2.7 million and 2.3 million had a chronic HBV and HCV coinfection, respectively [1,2]. It is estimated that hepatitis viruses caused 1.34 million deaths in 2015 while influenza viruses are responsible for 250,000 to 500,000 death globally every year. Despite the great achievements made in antiviral drug discovery during the past 50 years, there is still no effective antiviral drug for over 200 infectious diseases [2]. The increasing emergence of drug-resistant viral strains urgently requires the development of new antiviral drugs with novel action mechanisms. Particularly, broad-spectrum antiviral drugs are needed to combat multiple viral infectious diseases since most of the current antiviral drugs are effective to only certain viral strains [2].

**Figure 1:**
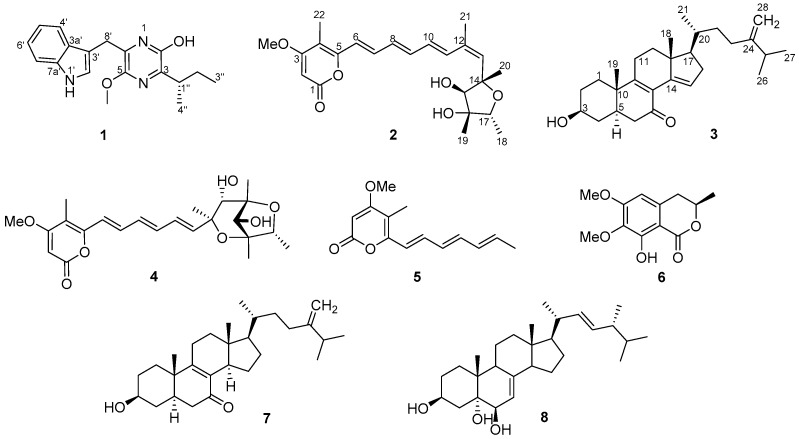
Structures of compounds **1**–**8** from *Penicillium* sp. IMB17-046.

Marine fungi are prolific sources of new structurally diverse compounds, and have yielded more than 1000 new metabolites, many of them displaying interesting biological activities, such as antibacterial, antifungal, antiviral, cytotoxic, and antiprotozoal properties [3,4]. During our ongoing program screening for new bioactive natural products from marine-derived microorganisms [5,6,7,8,9], the extracts from the solid cultures of the marine-derived strain *Penicillium* sp. IMB17-046 were shown to possess inhibitory activity against HIV-1 replication and antibacterial activities against Gram-negative bacteria. Further chemical investigation of the extracts led to the identification of a new pyrazine congener, trypilepyrazinol (**1**), a new α-pyrone polyketide, (+)-neocitreoviridin (**2**), and a new ergostane-type sterol, 3β-hydroxyergosta-8,14,24(28)-trien-7-one (**3**), together with the known epiisocitreoviridinol (**4**) [10], citreoviripyrone B (**5**) [11], kigelin (**6**) [12], 3β-hydroxyergosta-8,24(28)-dien-7-one (**7**) [13], and (22*E*,24*R*)-24-methyl-5α-cholesta-7,22-dien-3β,5,6β-triol (**8**) [14] (Figure 1). Herein, we describe the isolation, structural elucidation, and antiviral and antibacterial properties of the new compounds.

## 2. Results and Discussion

**Figure 2:**
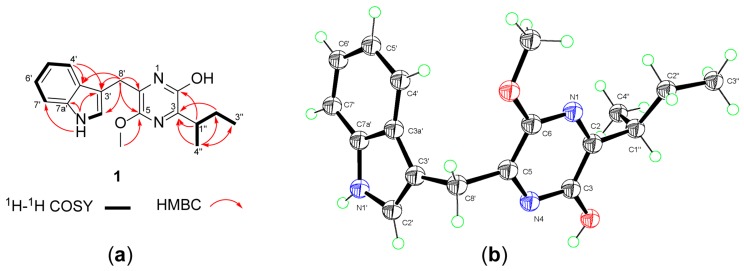
(**a**) The ^1^H–^1^H COSY and key HMBC correlations of **1**. (**b**) X-ray crystallographic structure of **1**.

Compound **1** was isolated as a colorless plate crystal. Its molecular formula was determined to be C_18_H_21_N_3_O_2_ by HRESIMS, indicating 10 degrees of unsaturation. The IR spectrum showed the presence of the hydroxy or amino (3421 cm^−1^) and the aromatic ring (1615 cm^−1^) groups. The ^1^H-NMR spectrum in CDCl_3_ (Table 1) revealed three methyl signals comprising a methoxy (δ_H_ 3.98) and two aliphatic methyls (δ_H_ 1.20, d; 0.87, t), two methylenes (δ_H_ 4.13/4.10; 1.81/1.55), a methine (δ_H_ 3.15), four protons between δ_H_ 7.09 and 7.68 ascribed to an *ortho*-disubstituted aromatic ring, an isolated olefinic proton (δ_H_ 7.22), as well as an exchangeable proton (δ_H_ 8.04, H-1′). The ^13^C-NMR and DEPT spectra showed 18 carbon signals, including three methyls, two methylenes, five sp^2^ one aliphatic methine, and seven non-protonated sp^2^ carbons. The COSY correlations of H-4′/H-5′/H-6′/H-7′ and H_3_-4′′/H-1′′/H-2′′a/H-2′′b/H_3_-3′′ confirmed the presence of the *ortho*-disubstituted aromatic ring and a *sec*-butyl fragment (Figure 2a). The HMBC cross-peaks of H_2_-8′ with C-2′, C-3′, and C-3a′; NH-1′ with C-3′, C-7′, and C-7a′; and H-4′ with C-3′ and C-3a′ suggested that the aromatic ring was present as an (indole-3-yl)methyl unit. In the ^13^C-NMR spectrum, four unassigned quaternary carbons resonated at δ_C_ 153.3, 149.8, 148.7, and 128.7. In addition, the remaining atoms required by the molecular formula included a hydrogen, an oxygen, and two nitrogens. By taking into consideration the unsaturation requirement, these data suggested the presence of a pyrazine ring in the molecule [15,16]. The HMBC correlations of H-1′′ with the carbons at δ_C_ 153.3 (C-2) and 149.8 (C-3) along with the correlation of H-4′′ with the carbon at δ_C_ 149.8 (C-3) located the *sec*-butyl at C-3 of the pyrazine ring. The HMBC correlations of H_2_-8′ with the carbons at δ_C_ 128.7 and δ_C_ 148.7 along with the correlation of the methoxy protons (δ_H_ 3.98) with the carbon at δ_C_ 148.7 suggested that the (indole-3-yl)methyl unit and the methoxy group were attached to the carbons at δ_C_ 128.7 and δ_C_ 148.7, respectively. However, these correlations did not allow for distinction of 2,5- versus 2,6-dioxy substitutions and thus could not define the location of the methoxy group (C-5 or C-6). Furthermore, it was not possible to determine whether the C-2 of the pyrazine ring was enolized based on the NMR data [15,16]. Therefore, compound **1** was subjected to X-ray crystallographic analysis using Cu Kα radiation (Figure 2b), which unambiguously established the structure as **1** with an enol group at C-2. The small values of the Flack parameter 0.02 (8) [17] and the Hooft parameter 0.04 (7) [18] in the final refinement of the crystallographic data allowed for assignment of the absolute configuration of **1** as 1′′*S*. Therefore, the structure of **1** was determined as (*S*)-6-((1*H*-indol-3-yl)methyl)-3-(*sec*-butyl)-5-methoxypyrazin-2-ol. According to the proposed biosynthetic pathway for natural pyrazines [19,20], compound **1** was probably derived from the precursors tryptophan and isoleucine and was thereby assigned the trivial name trypilepyrazinol.

**Figure 3:**
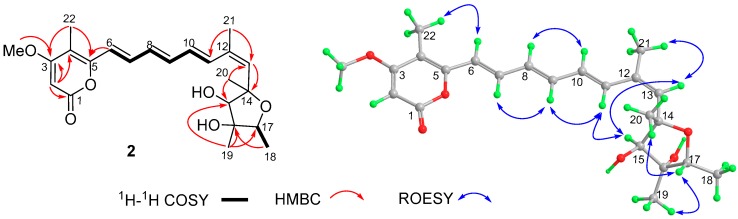
The ^1^H–^1^H COSY and the key HMBC and ROESY correlations of **2**.

**Figure 4:**
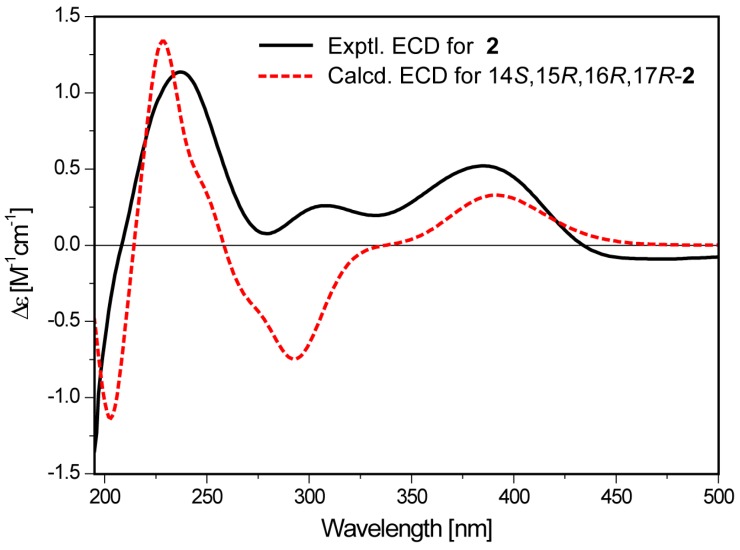
Comparison of the calculated and experimental ECD spectra of **2**.

Compound **2** was obtained as yellow needles with a molecular formula C_23_H_30_O_6_ as suggested by HRESIMS. The ^1^H-NMR spectrum of **2** displayed the signals for six coupled olefinic protons at δ_H_ 6.35–7.18, two isolated olefinic proton singlets at δ_H_ 5.51 (H-2) and 5.75 (H-13), two oxygenated methine protons at δ_H_ 4.02 (s, H-15) and 3,84 (q, H-17), five methyls at δ_H_ 1.20–1.96, and a methoxy group at *δ*_H_ 3.83 (Table 1). The ^13^C-NMR and DEPT spectra in CDCl_3_ showed the presence of one ester carbonyl, four nonprotonated sp^2^, and two nonprotonated oxygen-bearing sp^3^ carbons in addition to the methyl and methine signals mentioned above. The molecular formula, UV, and NMR data of **2** were similar to those of citreoviridin obtained from *Aspergillus terreus* [21] and other *Penicillium* strains (Table S1) [11,22,23]. Interestingly, compound **2** has a positive optical rotation ([α]${}_{D}^{20}$ +67.3 in MeOH), opposite to that of citreoviridin ([α]${}_{D}^{20}$ −105 in MeOH and −107.8 in CHCl_3_) [24,25]. In addition, the resonances for H-11 and C-21 in **2** were deshielded by Δ*δ*_H_ +0.77 and Δ*δ*_C_ +9.2 ppm, respectively, in comparison with those of citreoviridin recorded in CDCl_3_, whereas H-13, H_3_-21, C-11, and C-13 in **2** were shielded by Δδ_H_ −0.16 and −0.08 and Δδ_C_ −7.5 and −2.3 ppm, respectively, indicating that compound **2** was the Δ^12(13)^ geometrical isomer of citreoviridin. The ROESY correlations of H-13/H_3_-21 and H-11/H-15 confirmed the *Z*-geometry of the 12,13-double bond (Figure 3). The geometries for other double bonds were established as *E*, in accordance with those of citreoviridin, by the large coupling constants (15.0 Hz) ^3^*J*_H-6,H-7_, ^3^*J*_H-8__,H-9_, and ^3^*J*_H-10,H-11_ and confirmed by the ROSEY correlations of H-7/H-9, H-8/H-10, and H-9/H-11. In addition, the relative configuration of the tetrahydrofuran ring was determined to be identical with that of citreoviridin by ROESY correlations. The calculated ECD spectrum for 14*S*,15*R*,16*R*,17*R*-**2** matched well with the experimental curve, indicating that **2** possessed the identical absolute configuration with citreoviridin (Figure 4). Consequently, the structure of **2** was determined as 12*Z*-citreoviridin and named (+)-neocitreoviridin.

The molecular formula of compound **3** was determined as C_28_H_42_O_2_ by HRESIMS. The IR spectrum displayed the absorption bands for the hydroxy (3394 cm^−1^) and the unsaturated ketone (1665 cm^−1^) groups. Analysis of the ^1^H-NMR data revealed the signals for two tertiary methyls at δ_H_ 0.80 (s, H_3_-18) and 1.14 (s, H_3_-19); three secondary methyls at δ_H_ 0.99 (d, H_3_-21), 1.03 (d, H_3_-27), and 1.04 (d, H_3_-26); two terminal olefinic protons at δ_H_ 4.73 (d, *J* = 1.8 Hz, H-28a) and 4.68 (d, *J* = 1.8 Hz, H-28b); and one oxymethine proton at δ_H_ 3.67 (H-3). The ^13^C-NMR and DEPT spectra revealed 28 carbon resonances that were ascribed to five methyls, 10 methylenes (with one olefinic carbon at δ_C_ 106.1), six methines (including a cone oxygenated carbon at δ_C_ 69.9 and one sp^2^ carbon at δ_C_ 126.6), and seven nonprotonated carbons (with one carbonyl at δ_C_ 197.5 and four sp^2^ carbons at δ_C_ 127.1, 141.3, 156.7, and 165.2). These NMR data were similar to those of ergosta-24(28)-ene-3-ol derivatives [13], suggesting an ergostane skeleton for **3**.

**Figure 5:**
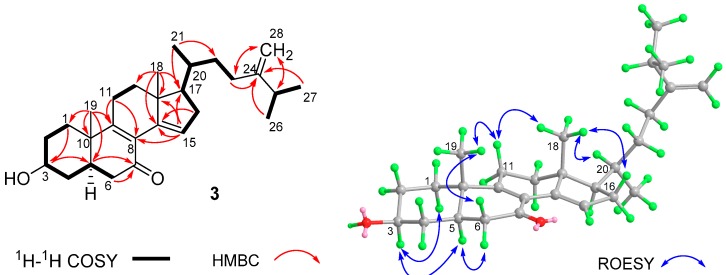
The ^1^H–^1^H COSY and key HMBC and ROESY correlations of **3**.

Analysis of the COSY data revealed the presence of four partial structures as illustrated by the bold lines in Figure 5. These partial structures were connected by the HMBC correlations from H_3_-19 to C-1, C-5, C-9, and C-10; from H_3_-18 to C-12, C-13, C-14, and C-17; and from H-15 to C-8 and C-13. The HMBC correlations of H_3_-26 and H_3_-27 with C-24 (δ_C_ 156.7) and H-25, H-23a, and H-23b with C-28 (δ_C_ 106.1) allowed the placement of one double bond at C-24 (C-28). The two carbon signals at δ_C_ 127.1 and 165.2 were allocated to C-8 and C-9 double bonds based on the HMBC correlations of H_3_-19 with C-9 (δ_C_ 165.2) and H-11a and 11b with C-8 (δ_C_ 127.1) and C-9. Further correlations of H_3_-18 with C-14 (δ_C_ 141.3) and H-16a and H-16b with C-14 and C-15 (δ_C_ 126.6) revealed the third double bond at C-14(C-15). HMBC correlations were observed from H-5, H-6a, and H-6b to the carbonyl carbon (δ_C_ 197.5), locating a ketone group at C-7. Finally, HMBC correlations of H_2_-1, H_2_-2, and H_2_-4 with C-3 (δ_C_ 69.9) allowed the assignment of an OH group at C-3, completing the full planar structure for **3**. The relative configuration of **3** was established by analysis of the coupling constants and ROESY data. The large coupling constants of *J*_H-2b,H-3_ (11.4 Hz) and *J*_H-3,H-4b_ (11.4 Hz) and the ROESY correlations of H-5 with H-3 and H-6a indicated the α- (axial) orientation for H-3, H-5, and H-6a. On the other hand, ROESY correlations of H_3_-19 with H-6b and H-11b and of H_3_-18 with H-11b and H-20 revealed these protons on the opposite side. Thus, the structure of **3** was determined as 3β-hydroxyergosta-8,14,24(28)-trien-7-one.

Compounds **1**–**8** were evaluated for their antiviral activities against HIV-1, influenza A virus (IAV), and HCV (Table 2). Of the tested compounds, compound **1** exhibited inhibitory activities against HIV-1 and HCV with IC_50_ values of 4.6 and 7.7 μM, respectively. Compound **2** showed significant inhibitory activity against IAV with an IC_50_ value of 3.6 μM as compared to 15.4 μM of the positive control ribavirin. Compound **3** showed anti-HIV activity with an IC_50_ of 3.5 μM and potent anti-IAV activity with an IC_50_ of 0.5 μM, 300-fold stronger than ribavirin. Compounds **4**–**6** were inactive against the above viruses at the concentration of 10 μM. It is interesting to note that the other ergostane derivatives, **7** and **8**, showed no detectable inhibitory activity against IAV (IC_50_ > 10 μM). A recent study by Ge et al. [26] showed that the ergostane derivative, (20*S*,24*R*)-3β,20-dihydroxyergostan-5(6)-en-7,16-dione (amotsterol D), which was effective against wild-type and multi-drug resistant HIV-1 in the low micromolar range, might target the host cell kinases PKM2, a rate-limiting enzyme of glycolysis, to inhibit replication of HIV-1. Host cell kinases are vital for the replication of a number of viruses and might be targets for broad-spectrum antivirals [27,28]. Due to the close structural similarity with amotsterol D, it was likely that compound **3** exerted broad-spectrum antiviral activity by the same target. *Helicobacter pylori* is a Gram-negative pathogen whose infection has been recognized as the causative factor of chronic gastritis, peptic ulceration, and gastric malignancies [29]. In the antibacterial assay, compounds **1** and **2** showed significant antibacterial activities against clinically isolated *H. pylori* (including the drug-sensitive strain G27 and the drug-resistant strain 159) with minimum inhibitory concentrations (MICs) of 1–16 μg/mL, whereas they were inactive against Gram-positive *Staphylococcus aureus* and *Bacillus subtilis* and Gram-negative *Pseudomonas aeruginosa* and *Klebsiella pneumoniae* (MIC > 128 μg/mL).

## 3. Materials and Methods

### 3.1. General Experimental Procedures

Optical rotations were measured using a Perkin-Elmer model 343 polarimeter (Perkin-Elmer Inc., Waltham, MA, USA). UV and ECD spectra were recorded on an Applied Photophysics Chirascan spectrometer (Applied Photophysics Ltd., Surrey, UK). IR spectra were measured using a Nicolet 5700 FT-IR microscope spectrometer (FT-IR microscope transmission) (Thermo Electron Corp., Madison, WI, USA). NMR spectra were acquired on a AVANCE III HD 600 MHz spectrometers (Bruker Corp., Karlsruhe, Germany) in CDCl_3_ with tetramethylsilane as an internal reference. ESIMS data were obtained using an Agilent 1100 LC/MSD with a G1956B single quadrupole mass spectrometer (Agilent Technologies, Ltd., Santa Clara, CA, USA). HRESIMS data were recorded using a Thermo LTQ Orbitrap XL mass spectrometer (Thermo Fisher Scientific, Waltham, MA, USA). Flash chromatography was performed on an Ez Purifier (Suzhou Lisure Science Co., Ltd., Suzhou, China). Column chromatography was carried out using silica gel (Qingdao Marine Chemical Inc, Qingdao, China) and Toyopearl gel HW-40F (Tosoh Co., Tokyo, Japan). HPLC separation was performed with a Shimadzu LC-20AP binary pump (Shimadzu Co., Kyoto, Japan) equipped with an SPD-M20A diode array detector using a Shiseido Capcell C18 MGII preparative (20 mm × 250 mm) or semi-preparative (10 mm × 250 mm) column.

### 3.2. Fungal Material

The fungus *Penicillium* sp. IMB17-046 was isolated from marine sediments collected from a mangrove swamp in Sanya, Hainan province, China. The strain was identified as a member of the genus *Penicillium* by morphological characteristics and sequence analysis. Its 18S and ITS-5.8S rDNA gene sequences (GenBank accession no. MK720046 and MK720045) showed the closest match with *P. decumbens* (GenBank KX553859) and *P. manginii* (GenBank MH858641) with 99.09% and 99.48% sequence similarities, respectively. The strain was deposited in the National Laboratory for Screening Microbial Drug, Institute of Medicinal Biotechnology, Chinese Academy of Medical Sciences.

### 3.3. Fermentation and Isolation

*Penicillium* sp. IMB17-046 was cultivated on potato dextrose agar (PDA) plates at 28 °C for 7 days. Agar cultures were cut into small pieces (about 1 cm^2^, one piece each) that were inoculated into 500 mL Fernbach flasks each containing 100 mL of the potato dextrose broth (PDB) medium (3 g of potato extract, 20 g of glucose in 1L of H_2_O) and cultured at 28 °C on a rotary shaker at 200 rpm for 5 days. The resulting seed cultures (each 10 mL) were transferred into 30 replicate 500 mL flasks each containing 100 mL of the rice medium (100 g of rice, 0.3 g of peptone in 100 mL H_2_O) for 4 weeks at 28 °C. The fungal rice cultures were sequentially extracted with EtOAc (3 × 6 L) and MeOH (3 × 6 L). After removing the organic solvent, the residual aqueous extracts were combined and partitioned in H_2_O, and extracted with petroleum ether (5 × 3 L) and EtOAc (5 × 3 L), successively, to give the corresponding extracts. The petroleum ether extracts (45 g) were subjected to silica gel column chromatography eluting with petroleum ether-EtOAc (15:1, 10:1, 4:1, 1:1, 0:1) to afford 12 fractions (F_1_−F_12_). Fraction F_4_ (800 mg) was separated on preparative reversed-phase (RP) C_18_ HPLC (68% MeCN–H_2_O, 10 mL/min) and further purified by semi-preparative HPLC (77% MeOH, 4 mL/min) to give **1** (36 mg). Fraction F_5_ (650 mg) was subjected to RP C18 flash chromatography with gradient elution of 20%−100% MeOH–H_2_O to afford six subfractions (F_5-1_–F_5-6_). Subfraction F_5-4_ (300 mg) was subjected to preparative RP C18 HPLC (80% MeCN–H_2_O, 10 mL/min) to yield **3** (20 mg). Fraction F_7_ (220 mg) was separated by preparative RP C18 HPLC (50% MeCN–H_2_O, 10 mL/min) and further purified by semi-preparative HPLC (70% MeOH–H_2_O, 4 mL/min) to yield **2** (20 mg). Purities of compounds **1** (>96%), **2** (>96%), and **3** (>97%) were determined by HPLC-DAD and verified by ^1^H-NMR analysis.

*Trypilepyrazinol* (**1**): Colorless plate crystal; [α]${}_{D}^{20}$ +8.6 (*c* 0.41, MeOH); UV (MeOH) *λ*_max_ (log*ε*) 221 (4.68), 281 (3.75), 325 (3.85), 368 (3.56) nm; ECD (*c* 2.0 × 10^−4^ M, MeOH) *λ*_max_ (Δ*ε*) 206 (−0.49), 237 (+0.14), 265 (−0.04), 324 (+0.37) nm; IR *v*_max_ 3421, 2966, 2936, 1642, 1615, 1520, 1467, 1413, 1167, 1023, and 744 cm^−1^; ^1^H-NMR (CDCl_3_, 600 MHz) and ^13^C-NMR (CDCl_3_, 150 MHz), Table 1; HRESIMS: *m/z* 312.1696 [M + H]^+^ (calcd for C_18_H_22_N_3_O_2_, 312.1707).

*(+)-Neocitreoviridin* (**2**): Yellow powder; [α]${}_{D}^{20}$ +67.3 (*c* 0.4, MeOH); UV (MeOH) *λ*_max_ (log*ε*) 235 (4.06), 291 (4.30), 390 (4.50) nm; ECD (*c* 6.2 × 10^−4^ M, MeOH) *λ*_max_ (Δ*ε*) 237 (+1.14), 308 (+0.26), 385 (+0.52) nm; IR *v*_max_ 3406, 2934, 1686, 1624, 1590, 1537, 1455, 1406, 1251, 1094, 995, and 803 cm^−1^; ^1^H-NMR (CDCl_3_, 600 MHz) and ^13^C-NMR (CDCl_3_, 150 MHz), Table 1; HRESIMS: *m/z* 403.2106 [M + H]^+^ (calcd for C_23_H_31_O_6_, 403.2115).

*3β-Hydroxyergosta-8,14,24(28)-trien-7-one* (**3**): White, amorphous powder; [α]${}_{D}^{20}$ −4.0 (*c* 0.43, MeOH); UV (MeOH) *λ*_max_ (log*ε*) 297 (3.43) nm; ECD (*c* 6.1 × 10^−4^ M, MeOH) *λ*_max_ (Δ*ε*) 211 (−6.78), 247 (+1.44), 285 (−1.52), 336 (+0.35) nm; IR *v*_max_ 3394, 2925, 1665, 1650, 1467, 1377, and 1041 cm^−1^; ^1^H-NMR (CDCl_3_, 600 MHz) and ^13^C-NMR (CDCl_3_, 150 MHz), Table 1; HRESIMS: *m/z* 411.3258 [M + H]^+^ (calcd for C_28_H_43_O_2_, 411.3258).

### 3.4. Crystallographic Analysis of **1**

A colorless plate crystal of **1** was obtained from CHCl_3_-MeOH (9:1 *v*/*v*) via slow evaporation. The crystal data were obtained at 293 K using Cu K radiation (1.54184 Å) on an Oxford Diffraction Gemini Ultra CCD diffractometer. The structure was solved by direct methods (SHELXS-97) and refined with the SHELXL-97 refinement package using Least Squares minimization [30]. Crystal data of 1 was deposited in the Cambridge Crystallographic Data Centre with the deposition number CCDC 1935589. CCDC 1935589 contains the supplementary crystallographic data for this paper. These data can be obtained free of charge via http://www.ccdc.cam.ac.uk/conts/retrieving.html (or from the CCDC, 12 Union Road, Cambridge CB2 1EZ, UK; Fax: +44 1223 336033; E-mail: deposit@ccdc.cam.ac.uk).

Crystal data of 1: 2(C_18_H_21_N_3_O_2_), M = 622.75, monoclinic, space group C2, a = 21.9518(3) Å, b = 6.1592(1) Å, c = 25.3607(3) Å, α = 90°, β = 102.5169(13)°, γ = 90°, V = 3347.43(8) Å3, Z = 4, μ(Cu Kα) = 0.659, Dcalc = 1.236 g/cm^3^; 13396 reflections measured in the range of 4.0940° ≤ θ ≤ 66.5790°, 5664 independent reflections, *R*_int_ =0.0215, 424 parameters, 1 restraints. The final indices were *wR*_2_ = 0.1076, *R*_1_ = 0.0372 [I > 2σ(*I*)]. The goodness of fit on F^2^ was 1.036. Flack parameter x = 0.02(8). The Hooft parameter y = 0.04(7).

### 3.5. ECD Calculation of (14S,15R,16R,17R)-**2**

A conformation search was performed on Spartan 14 software (version 1.1.0, Wavefunction Inc. 2014, Irvine, CA, USA) using the MMFF94 molecular mechanics force field. The obtained conformers within a 4 kcal/mol upper energy limit were geometry optimized at the B3LYP/6-31+G (d,p) level in gas using the Gaussian 09 program [31]. The harmonic vibrational frequencies were calculated at the same level to provide their free energy values (ΔG). The low-energy conformers with ΔG ≤ 4.0 kcal/mol were subjected to further geometry optimization and frequency calculation at the M06-2X/6-311+G(d,p) level with the polarizable continuum model (PCM) in MeOH. The equilibrium population of each conformer at 298.15 K was calculated according to the ΔG obtained by the second-round optimization at the m06-2x/6-311+G(d,p) level. Energies of the conformers with Boltzmann distribution (>1%) were calculated using the TDDFT methodology (NStates = 65) at the CAM-B3LYP/TZVP level. The ECD spectrum of each conformer was generated by the SpecDis program [32] using a Gaussian function band width σ = 0.30 eV. The calculated ECD spectrum for (14*S*,15*R*,16*R*,17*R*)-**2** was obtained by averaging the calculated data of each conformer according to their Boltzmann population.

### 3.6. Anti-HIV Assay

Anti-HIV activities were evaluated according to a procedure described previously [33]. Briefly, 293T cells were co-transfected with the vector pNL-luc-E- containing a full-length HIV-1 proviral DNA with a firefly luciferase gene and the vesicular stomatitis virus glycoprotein-expressing vector pHCMV-G. After cultivation for 48 h, the vesicular stomatitis virus glycoprotein (VSV-G) pseudotyped HIV-1 viruses were harvested by filtration. SupT1 cells (1 × 10^5^) were infected with the VSV-G-HIV virus (multiplicity of infection, MOI = 1) and treated with the test compounds in triplicate. Efavirenz was used as a positive control. After 48 h inoculation, the SupT1 cells were lysed and the inhibition rate was determined using a firefly Luciferase Assay System (Promega). The concentration of compounds inhibiting 50% viral replication (IC_50_) was calculated by Origin 8.0 (OriginLab Co. Northampton, MA, USA).

### 3.7. Anti-Influenza A Virus Assay

Anti-influenza A virus assays were performed by using the cell-based high-throughput approach [34]. Briefly, 293T-Gluc cells were treated with test compounds and incubated for 2 h prior to infection. Then, the cells were infected with influenza A/WSN/33 (H1N1) viruses with an MOI of 0.3. After a further 24 h incubation, the cell supernatant was collected and measured for *Gaussia* luciferase activity. Ribavirin was used as a positive and negative control.

### 3.8. Anti-HCV Assay

Anti-HCV assays were carried out as described previously [5].

### 3.9. Cytotoxicity Assay

Cytotoxicity of compounds were assayed for the uninfected 293T-Gluc, SupT1, and Huh7.5 cells. 293T-Gluc and Huh7.5 cells were cultured in Dulbecco’s modified Eagle’s medium supplemented with 10% fetal bovine serum (FBS, Invitrogen). SupT1 cells were cultured in RPMI1640 medium supplemented with 10% FBS. Various concentrations of test compounds (1 μL each well) were added to the 293T-Gluc and SupT1 cells (1 × 10^5^ cells/well) in 96-well plates. After incubation at 37 °C for 48 h, 10 μL of CCK-8 reagent was added to the cells and they were incubated for another 4 h. Then, the absorbance of each well was recorded at 450 nm. A cytotoxicity assay for Huh7.5 cells was carried out using the MTT method as described previously [5]. The 50% cytotoxicity concentration (CC_50_) was calculated by Origin 8.0 software. 

### 3.10. Antibacterial Assay

The antibacterial activities against *H. pylori*, *S. aureus*, *B. subtilis*, *P. aeruginosa,* and *K. pneumonia* were assayed by examining the MIC using the broth micro-dilution method as previously described [5,35].

## 4. Conclusions

In summary, three new natural products, trypilepyrazinol (**1**), (+)-neocitreoviridin (**2**), and 3β-hydroxyergosta-8,14,24(28)-trien-7-one (**3**) were identified from the solid cultures of the mangrove-derived *Penicillium* sp. IMB17-046. Compounds **1** and **3** showed broad-spectrum antiviral properties against different types of viruses while **2** displayed significant antibacterial activity against *H. pylori* as well as an anti-IAV effect. Trypilepyrazinol (**1**) is characterized by a pyrazine motif. Pyrazine heterocycle is an important pharmacophore present as a basic scaffold in various clinical drugs with a wide range of pharmacological and therapeutic activities, such as antitumor, anti-inflammatory, antithrombotic, anti-diabetic, and anti-tubercular [36,37]. However, natural products containing a pyrazine moiety are relatively rare [19]. To the best of our knowledge, trypilepyrazinol (**1**) is the first example of natural pyrazines exhibiting broad-spectrum antiviral activities and antibacterial activity against *H. pylori*.

## Figures and Tables

**Table 1:** NMR spectroscopic data for compounds **1**–**3** in CDCl_3_.^a^

No.	1		No.	2		3	
	δ_C_, Type	δ_H_, Mult. (*J* in Hz)		δ_C_, Type	δ_H_, Mult. (*J* in Hz)	δ_C_, Type	δ_H_, Mult. (*J* in Hz)
2	153.3, C		1	164.0, C		34.8, CH_2_	1.92, m; 1.46, m
3	149.8, C		2	88.6, CH_2_	5.51, s	31.3, CH_2_	1.94, m; 1.54, m
5	148.7, C		3	170.8, C		69.9, CH	3.67, dddd (11.4, 11.4, 4.8, 4.2)
6	128.7, C		4	108.0, C		37.3, CH_2_	1.75, m; 1.42, m
1′		8.04, s	5	154.5, C		38.8, CH	2.00, m
2′	123.5, CH	7.22, d (2.4)	6	119.0, CH	6.35, d (15.0)	42.1, CH_2_	2.37, dd (10.8, 16.8)
3′	111.4, CH						2.35, dd (10.8, 4.8)
3a′	127.3, C		7	136.0, CH	7.18, dd (10.8, 15.0)	197.5, C	
4′	119.3, CH	7.68, brd (7.8)	8	131.8, CH	6.40, dd (10.8, 15.0)	127.1, C	
5′	119.8, CH	7.09, ddd (7.8, 7.2, 1.2)	9	139.0, CH	6.57, dd (10.8, 15.6)	165.2, C	
6′	122.4, CH	7.18, ddd (7.8, 7.2, 1.2)	10	129.9, CH	6.31, dd (10.8, 15.0)	38.0, C	
7′	111.3, CH	7.34, brd (7.8)	11	133.2, CH	7.09, d (15.6)	24.2, CH_2_	2.54, dd (20.4, 5.4)
7a′	136.3, C						2.41, ddd (20.4, 12.0, 6.0)
8′	24.9, CH_2_	4.13, d (15.6)	12	132.0, C		36.0, CH_2_	2.10, dd (12.0, 6.0); 1.47, m
		4.10, d (15.6)	13	138.7, CH	5.75, s	45.7, C	
1′′	36.2, CH	3.15, sextet (6.6)	14	84.5, C		141.3, C	
2′′	28.0, CH_2_	1.81, m	15	86.2, CH	4.02, s	126.6, CH	6.46, brs
		1.55, m	16	81.0, C		36.9, CH_2_	2.48, ddd (16.2, 7.2, 3.0)
3′′	12.2, CH_3_	0.87, t (7.2)					2.19, dd (16.2, 6.0)
4′′	18.4, CH_3_	1.20, d (6.6)	17	77.6, CH	3.84, q (6.6)	55.8, CH	1.52, m
OMe	54.4, CH_3_	3.98, s	18	12.3, CH_3_	1.20, d (6.6)	15.5, CH_3_	0.80, s
			19	17.4, CH_3_	1.23, s	17.6, CH_3_	1.14, s
			20	20.7, CH_3_	1.40, s	34.0, CH	1.65, m
			21	22.6, CH_3_	1.85, s	19.0, CH_3_	0.99, d (6.6)
			22	8.9, CH_3_	1.96, s	34.5, CH_2_	1.61, m; 1.22, m
			23			30.9, CH_2_	2.13, m; 1.93, m
			24			156.7, C	
			25			33.8, CH	2.24, m
			26			22.0, CH_3_	1.04, d (7.2)
			27			21.9, CH_3_	1.03, d (7.2)
			28			106.1, CH_2_	4.73, d (1.8); 4.68, d (1.8)
			OMe	56.3, CH_3_	3.83, s		

^a^ The assignments were based on 2D-NMR (^1^H–^1^H COSY, HSQC, and HMBC) experiments.

**Table 2:** Antiviral (IC_50_ and CC_50_, μM) and antibacterial (MIC, μg/mL) activities of compounds **1**–**3**, **7**, and **8**.

Compound	HIV-1		IAV		HCV		*H. pylori* (MIC, μg/mL)	
	IC_50_ (μM)	CC_50_ (μM)	IC_50_ (μM)	CC_50_ (μM)	IC_50_ (μM)	CC_50_ (μM)	G27	159
**1**	4.6 ± 0.3	44.3 ± 1.6	20.4 ± 0.3	76.7 ± 4.6	7.7 ± 0.2	116.1 ± 4.9	4	16
**2**	>10	>100	3.6 ± 0.2	>100	NT	NT	4	1
**3**	3.5 ± 0.8	51.2 ± 3.5	0.5 ± 0.02	>100	NT	NT	NT	NT
**7**	>10	>100	>10	>100	NT	NT	NT	NT
**8**	6.2 ± 0.2	26.0 ± 0.2	>10	>100	NT	NT	NT	NT
Efavirenz	0.0005 ± 0.0002	>100	NT	NT	NT	NT	NT	NT
Ribavirin	NT	NT	15.4 ± 0.9	>100	NT	NT	NT	NT
VX-950	NT	NT	NT	NT	0.05 ± 0.03	25.8 ± 3.4	NT	NT
Metronidazole	NT	NT	NT	NT	NT	NT	1	16

HIV: human immunodeficiency virus; IAV: influenza A virus; HCV: hepatitis C virus; MIC: minimum inhibitory concentration; NT: Not tested.3. Materials and Methods.

## Supplementary Materials

The Supplementary Materials are available online.
