# Supplementary material for: Broad-Spectrum Antiviral Natural Products from the Marine-Derived Penicillium sp. IMB17-046
Source: Molecules. 2019 Aug 2;24(15):2821. doi: 10.3390/molecules24152821 (PMC6696147; doi:10.3390/molecules24152821)
Supplement: Supplementary file 1 [file molecules-24-02821-s001.pdf]

Broad-Spectrum Antiviral Natural Products from the Marine-derived *Penicillium* sp. IMB17-046

Jiao Li [1,†], Yujia Wang [1,†], Xiaomeng Hao [1], Shasha Li [1], Jia Jia [2], Yan Guan [1], Zonggen Peng [1], Hongkai Bi [2], Chunling Xiao [1], Shan Cen [1], and Maoluo Gan [1,*]

[1] Institute of Medicinal Biotechnology, Chinese Academy of Medical Sciences and Peking Union Medical College, Beijing 100050, China; jiaoli930911@126.com (J.L.); yujia.wang@imb.pumc.edu.cn (Y.W.); xiaomhao@163.com (X.H.); veraleehp@163.com; guanyan20@163.com (Y.G.); pumcpzg@126.com (Z.P.); shancen@imb.pumc.edu.cn (S.C.); xiaocl318@163.com (C.X.)

[2] Department of Pathogen Biology, Jiangsu Key Laboratory of Pathogen Biology, Nanjing Medical University, Nanjing, Jiangsu, P. R. China; jiajia@njmu.edu.cn; hkbi@njmu.edu.cn (H.B.)

* Correspondence: ganml@imb.pumc.edu.cn; Tel.: +86-10-63165277

† These authors contributed equally to this work.

Supporting Information

Contents

Physical and Spectroscopic data of Compounds 4-8 3
Table S1. ^{13}C (150 MHz) and ^{1}H (600 MHz) NMR data for (+)-neocitreoviridin (**2**) and (−)-citreoviridin 5
Figure S1. The (+)-HRESIMS spectrum of trypilepyrazinol (**1**). 6
Figure S2. The IR spectrum of trypilepyrazinol (**1**). 7
Figure S3. The ^{1}H NMR spectrum of trypilepyrazinol (**1**) in $CDCl_3$ (600 MHz). 8
Figure S4. The ^{13}C NMR spectrum of trypilepyrazinol (**1**) in $CDCl_3$ (150 MHz) 9
Figure S5. The DEPT spectrum of trypilepyrazinol (**1**) in $CDCl_3$ (150 MHz). 10
Figure S6. The ^{1}H-^{1}H COSY spectrum of trypilepyrazinol (**1**) in $CDCl_3$ (600 MHz). 11
Figure S7. The HSQC spectrum of trypilepyrazinol (**1**) in $CDCl_3$ (600 MHz). 12
Figure S8. The HMBC spectrum of trypilepyrazinol (**1**) in $CDCl_3$ (600 MHz). 13
Figure S9. The (+)-HRESIMS spectrum of (+)-neocitreoviridin (**2**). 14
Figure S10. The IR spectrum of (+)-neocitreoviridin (**2**). 15
Figure S11. The ^{1}H NMR spectrum of (+)-neocitreoviridin (**2**) in $CDCl_3$ (600 MHz). 16
Figure S12. The ^{13}C NMR spectrum of (+)-neocitreoviridin (**2**) in $CDCl_3$ (150 MHz). 17
Figure S13. The ^{1}H-^{1}H COSY spectrum of (+)-neocitreoviridin (**2**) in $CDCl_3$ (600 MHz) 18
Figure S14. The HSQC spectrum of (+)-neocitreoviridin (**2**) in $CDCl_3$ (600 MHz). 19
Figure S15. The HMBC spectrum of (+)-neocitreoviridin (**2**) in $CDCl_3$ (600 MHz) 20
Figure S16. The ROESY spectrum of (+)-neocitreoviridin (**2**) in $CDCl_3$ (600 MHz). 21
Figure S17. The (+)-HRESIMS spectrum of 3*β*-hydroxyergosta-8,14,24(28)-trien-7-one (**3**). 22
Figure S18. The IR spectrum of 3*β*-hydroxyergosta-8,14,24(28)-trien-7-one (**3**). 23
Figure S19. The ^{1}H NMR spectrum of 3*β*-hydroxyergosta-8,14,24(28)-trien-7-one (**3**) in $CDCl_3$ (600 MHz). 24
Figure S20. The ^{13}C NMR spectrum of 3*β*-hydroxyergosta-8,14,24(28)-trien-7-one (**3**) in $CDCl_3$ (150 MHz). 25
Figure S21. The DEPT spectrum of 3*β*-hydroxyergosta-8,14,24(28)-trien-7-one (**3**) in $CDCl_3$ (150 MHz). 26
Figure S22. The ^{1}H-^{1}H COSY spectrum of 3*β*-hydroxyergosta-8,14,24(28)-trien-7-one (**3**) in $CDCl_3$ (600 MHz). 27
Figure S23. The HSQC spectrum of 3*β*-hydroxyergosta-8,14,24(28)-trien-7-one (**3**) in $CDCl_3$ (600 MHz). 28
Figure S24. The HMBC spectrum of 3*β*-hydroxyergosta-8,14,24(28)-trien-7-one (**3**) in $CDCl_3$ (600 MHz). 29
Figure S25. The ROESY spectrum of 3*β*-hydroxyergosta-8,14,24(28)-trien-7-one (**3**) in $CDCl_3$ (600 MHz) 30
Figure S26. The ^{1}H NMR spectrum of epiisocitreoviridinol (**4**) in $CDCl_3$ (600 MHz). 31
Figure S27. The ^{1}H NMR spectrum of citreoviripyrone B (**5**) in $CDCl_3$ (600 MHz). 32
Figure S28. The ^{1}H NMR spectrum of kigelin (**6**) in $CDCl_3$ (600 MHz). 33
Figure S29. The ^{1}H NMR spectrum of 3*β*-hydroxyergosta-8,24(28)-dien-7-one (**7**) in $CDCl_3$ (600 MHz). 34
Figure S30. The ^{1}H NMR spectrum of (22*E*,24*R*)-24-methyl-5α-cholesta-7,22-dien-3*β*,5,6*β*-triol (**8**) in DMSO-d_6 (600 MHz). 35
Figure S31. Optimized conformers (≥ 1%) of 14*S*,15*R*,16*R*,17*R*-**2** at the M06-2X/6-311+G(d,p) level in MeOH 36
Table S2. Boltzmann populations of the identified conformers for 14*S*,15*R*,16*R*,17*R*-**2** 36
Figure S32. Comparison of the experimental and calculated ECD and UV spectra of **2** 37
Table S3. Calculated ECD data for conformers **2-C1** and **2-C2** at the Cam-B3LYP/TZVP//M06-2X/6-311+G(d,p) level in MeOH 38
Table S4. Calculated ECD data for conformers **2-C3** and **2-C4** at the Cam-B3LYP/TZVP//M06-2X/6-311+G(d,p) level in MeOH 39
Figure S33. The HPLC analysis of **1–3** 40

Physical and Spectroscopic data of Compounds 4-8

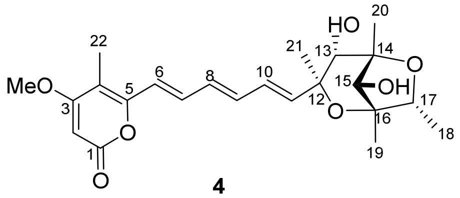

4

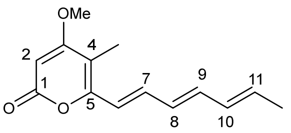

5

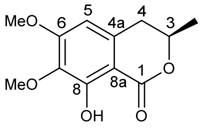

6

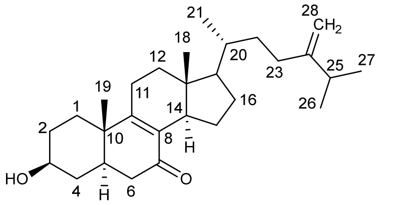

7

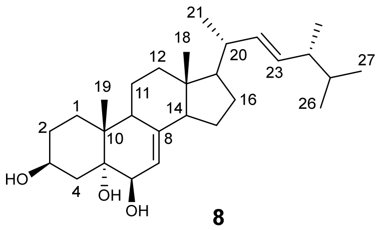

8

Epiisocitreoviridinol (**4**): yellow needles; $[\alpha]^{20}_{D}$ + 64.0 (*c* 0.13, MeOH); UV (MeOH) λ_{max} 370(logε4.76), 274(4.70)nm; ECD (*c* 0.3 $\times 10^{-3}$ M, MeOH) λ_{max} ($\Delta\varepsilon$) 219 (+7.70) nm, 269 (-11.29), 368 (+4.24) nm; IR ν_{max} 3440, 1705, 1409, 1250, 1092, 1406, 1250, 1093,1016, and 813 cm^{-1}; ^{1}H-NMR ($CDCl_3$, 600 MHz) δ 5.50 (s, H-2), 6.36, (d, *J* = 15.0Hz, H-6), 7.18, (dd, *J* = 15.0, 10.8Hz, H-7), 6.34 (dd, *J* = 15.0, 10.8Hz, H-8), 6.44 (dd, *J* = 15.0, 10.8Hz, H-9), 6.35 (dd, *J* = 15.6, 10.8Hz, H-10), 5.96 (d, *J* = 15.6Hz, H-11), 3.58 (d, *J* = 10.8Hz, H-13), 4.26 (brs, H-15), 4.09 (q, *J* = 6.6Hz, H-17), 1.20 (d, *J* = 6.6Hz, H-18), 1.34 (s, H-19), 1.37 (s, H-20), 1.27 (s, H-21), 1.97 (s, H-22), 3.84 (s, OMe-3), 3.30 (d, *J* = 10.8Hz, OH-13); ^{13}C NMR ($CDCl_3$, 150 MHz) 163.7 (C-1), 88.8 (C-2), 170.6 (C-3), 108.1 (C-4), 154.3 (C-5), 119.4 (C-6), 135.5 (C-7), 131.6 (C-8), 137.0 (C-9), 128.1 (C-10), 146.5 (C-11), 78.2 (C-12), 79.6 (C-13), 82.6 (C-14), 75.2 (C-15), 83.0 (C-16), 80.2 (C-17), 13.2 (C-18), 17.3 (C-19), 18.6 (C-20), 26.5 (C-21), 8.9 (C-22), 56.2 (OMe-3); HRES-MS: m/z 403.2098 [M+H]+ (calcd for $C_{23}H_{30}O_7$, 403.2121).

Citreviripyrone B (**5**): yellow powder; UV (MeOH, HPLC) λ_{max} 355nm; ^{1}H-NMR ($CDCl_3$, 600 MHz) δ5.50 (s, H-2), 6.32 (d, *J* = 15.0 Hz, H-6), 7.18 (dd, *J* = 15.0, 11.0 Hz, H-7), 6.25 (dd, *J* = 15.0, 11.0 Hz, H-8), 6.45 (dd, *J* = 15.0, 10.6 Hz, H-9), 6.15 (dd, *J* = 15.0, 10.6 Hz, H-10), 5.90 (m, H-11), 1.84 (d, *J* = 6.0 Hz, H-12), 1.95 (s, H-13); ^{13}C NMR ($CDCl_3$, 150 MHz) δ163.9 (C-1), 88.7 (C-2), 170.8 (C-3), 107.6 (C-4), 154.8 (C-5), 118.4 (C-6), 136.4 (C-7), 129.2 (C-8), 138.6 (C-9), 131.7 (C-10), 133.8 (C-11), 18.7 (C-12), 9.0 (C-13); LC-MS *m/z* 233 $[M+H]^+$.

Kigelin (**6**): amorphous powder; UV (MeOH, HPLC) λ_{max} 269, 367 nm; ECD (*c* 0.1 $\times 10^{-3}$ M, MeOH) λ_{max} ($\Delta\varepsilon$) 216 (-1.78) nm, 244 (+0.45), 272 (-1.13), 310 (-0.37) nm; ^{1}H-NMR ($CDCl_3$, 600 MHz) δ4.69 (m, H-3), 2.89 (dd, *J* = 16.2, 10.2 Hz, H-4a), 2.85 (dd, *J* = 16.2, 4.8 Hz, H-4b), 6.28 (s, H-5), 1.52 (d, *J* = 6.6 Hz, Me-3), 3.88 (s, OMe-6), 3.92 (s, OMe-7), 11.15 (s, OH-8); ^{13}C NMR ($CDCl_3$, 150 MHz) δ169.8 (C-1), 75.8 (C-3), 34.7 (C-4), 102.0 (C-5), 158.4 (C-6), 135.3 (C-7), 156.2 (C-8), 102.8 (C-8a), 135.4 (C-4a), 20.7 (Me-3), 56.2 (OMe-6), 60.8 (OMe-7); LC-MS *m/z* 239 $[M+H]^+$.

3β-Hydroxyergosta-8,24(28)-dien-7-one (**7**): white powder; UV (MeOH, HPLC) λ_{max} 251 nm; ^{1}H-NMR ($CDCl_3$, 600 MHz) δ3.65 (m, H-3), 1.15 (m, H-17), 0.59 (s, H-18), 1.18 (s, H-19), 0.97 (d, *J* = 6.6 Hz, H-21), 1.55 (m, H-22a), 1.15 (m, H-22b), 2.23 (m, H-25), 1.03 (d, *J* = 6.6 Hz, H-26), 1.02 (d, *J* = 6.6 Hz, H-27), 4.72 (brs, H-28a), 4.66 (brs, H-28b), 1.14-1.60 (11H, H-1b, 4b, 12b, 15b, 16b, 17, 20, H_2-2, H_2-22), 1.68-2.46 (14H, H-1a, 4a, 5, 12a,14, 15a, 16a, 25, H_2-6, H_2-11, H_2-23); ^{13}C NMR (DMSO-d_6, 150 MHz) δ34.4 (C-1), 31.2 (C-2), 70.1 (C-3), 37.4 (C-4), 41.1 (C-5), 42.5 (C-6), 198.9 (C-7), 133.2 (C-8), 165.1

(C-9), 38.3 (C-10), 25.4 (C-11), 35.7 (C-12), 42.5 (C-13), 48.2 (C-14), 29.2 (C-15), 24.8 (C-16), 53.4 (C-17), 11.4 (C-18), 17.2 (C-19), 36.1 (C-20), 18.8 (C-21), 34.6 (C-22), 31.1 (C-23), 156.7 (C-24), 33.8 (C-25), 22.0 (C-26), 21.9 (C-27), 106.0 (C-28); LC-MS *m/z* 413 $[M+H]^+$。

(22*E*,24*R*)-24-Methyl-5α-cholesta-7,22-dien-3*β*,5,6*β*-triol (**8**): white powder; UV (MeOH, HPLC) λ_{max} 222, 262 nm; ^{1}H-NMR ($CDCl_3$, 600 MHz) δ3.76 (m, H-3), 3.37 (m, H-6), 5.08 (m, H-7), 0.54 (s, H-18), 0.91 (s, H-19), 1.00 (d, *J* = 6.6 Hz, H-21), 5.24 (dd, *J* = 15.0, 7.2 Hz, H-22), 5.17 (dd, *J* = 15.0, 8.4 Hz, H-23), 0.80 (d, *J* = 6.6 Hz, H-26), 0.89 (d, *J* = 6.6 Hz, H-27), 0.81 (d, *J* = 6.6 Hz, H-28), 1.20-1.60 (H-1, H-4, H-11, H-15, H-17, H-25), 1.80-2.02 (H-2, H-9, H-12, H-14, H-20, H-24), 4.51 (d, *J* = 4.5 Hz, OH-6), 4.22 (d, *J* = 5.0 Hz, OH-3), 3.60 (s, OH-5); ^{13}C NMR (DMSO-d_6, 150 MHz) δ21.3 (C-1), 40.2 (C-2), 66.0 (C-3), 31.2 (C-4), 74.4 (C-5), 72.1 (C-6), 119.4 (C-7), 139.6 (C-8), 42.3 (C-9), 36.6 (C-10), 32.5 (C-11), 39.0 (C-12), 43.0 (C-13), 54.2 (C-14), 22.6 (C-15), 27.8 (C-16), 55.3 (C-17), 12.1 (C-18), 17.7 (C-19), 40.0 (C-20), 21.0 (C-21), 135.4 (C-22), 131.4 (C-23), 42.0 (C-24), 32.5 (C-25), 19.8 (C-26), 19.5 (C-27), 17.3 (C-28); LC-MS *m/z* 445 $[M+H]^+$.

Table S1. ^{13}C (150 MHz) and ^{1}H (600 MHz) NMR data for (+)-neocitreoviridin (**2**) and (−)-citreoviridin

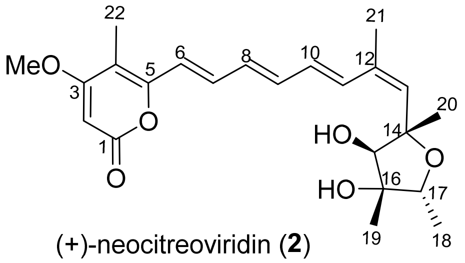

(+)-neocitreoviridin (**2**)

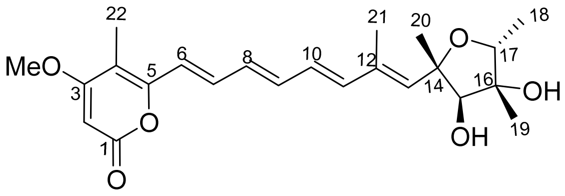

(−)-citreoviridin

No.	**2** (CDCl$_3$)		citreoviridin (CDCl$_3$)[a]		$\Delta\delta_H$	$\Delta\delta_C$
	δ_H, mult. (J in Hz)	δ_C, type	δ_H, mult. (J in Hz)	δ_C		
1		164.0		163.7	-	0.3
2	5.51, s	88.6	5.49, s	88.6	0.02	0
3		170.8		170.6	-	0.2
4		108.0		107.7	-	0.6
5		154.5		154.6	-	-0.1
6	6.35, d (15.0)	119.0	6.32, d (15.0)	118.7	0.03	0.3
7	7.18, dd (10.8, 15.0)	136.0	7.19, dd (15.0, 11.0)	136.0	-0.01	0
8	6.40, dd (10.8, 15.0)	131.8	6.36, dd (15.0, 10.3)	131.2	0.04	0.6
9	6.57, dd (10.8, 15.6)	139.0	6.50, dd (15.0, 10.3)	138.5	0.07	0.5
10	6.31, dd (10.8, 15.0)	129.9	6.26, dd (15.0, 10.3)	127.3	0.05	2.6
11	7.09, d (15.6)	133.2	6.32, d (15.0)	140.7	**0.77**	**-7.5**
12		132.0		134.3	-	**-2.3**
13	5.75, s	138.7	5.91, s	141.0	**-0.16**	**-2.3**
14		84.5		84.1	-	0.4
15	4.02, s	86.2	4.00, s	85.9	0.02	0.3
16		81.0		80.8	-	0.2
17	3.84, q (6.6)	77.6	3.83, q (6.6)	77.6	0.01	0
18	1.20, d (6.6)	12.3	1.18, d (6.6)	12.3	0.02	0
19	1.23, s	17.4	1.21, s	17.2	0.02	0.2
20	1.40, s	20.7	1.37, s	21.3	0.03	-0.6
21	1.85, s	22.6	1.93, s	13.4	**-0.08**	**9.2**
22	1.96, s	8.9	1.93, s	8.8	0.03	0.1
OMe	3.83, s	56.3	3.83, s	56.1	0	0.2

[a] The NMR data were cited from ref. 1.

References

1. Asai, T.; Luo, D.; Yamashita, K.; Oshima, Y. Org. Lett. 2013, 15, 1020-1023

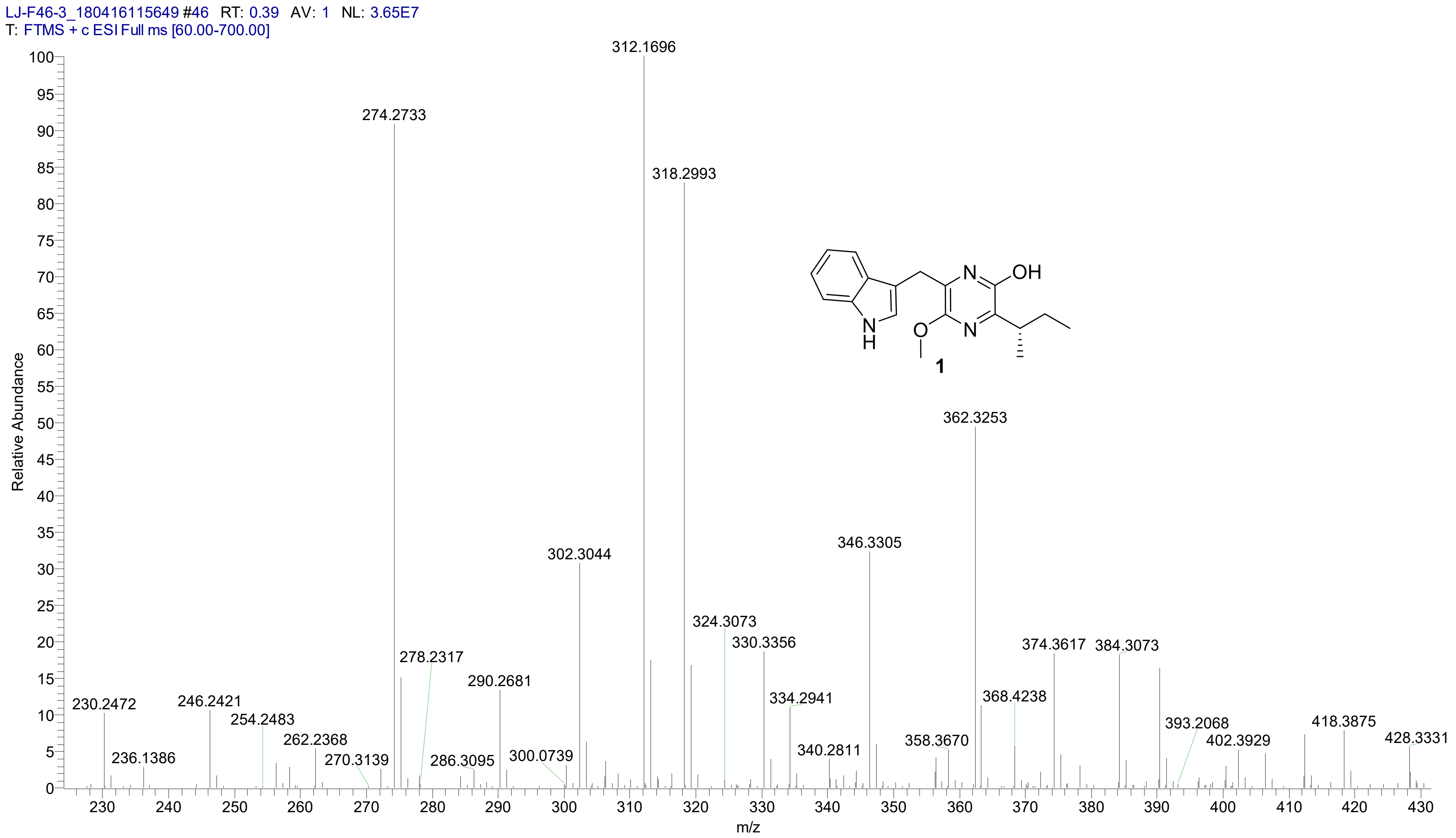

Figure S1. The (+)-HRESIMS spectrum of trypilepyrazinol (**1**).

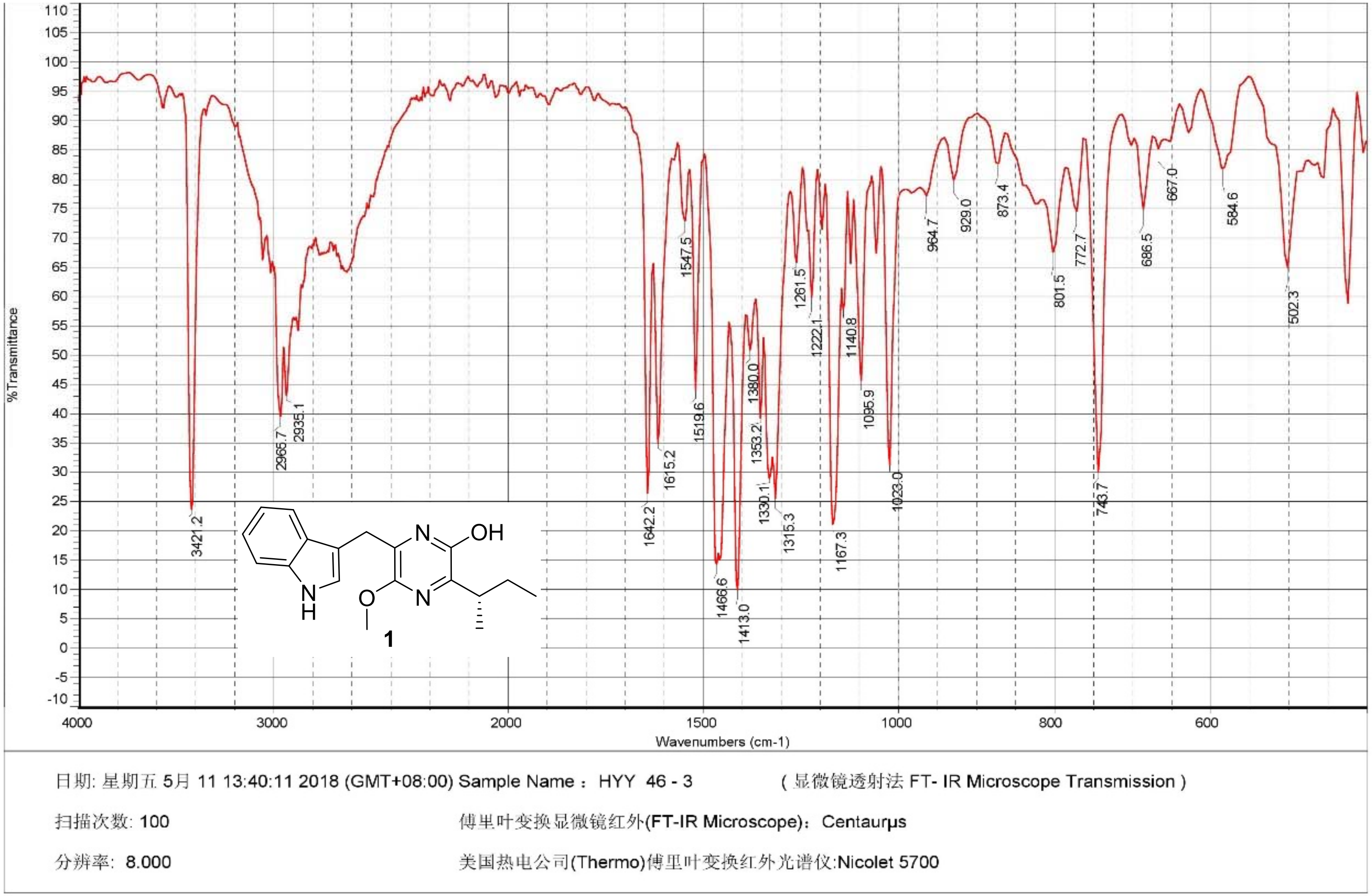

Figure S2. The IR spectrum of trypilepyrazinol (**1**).

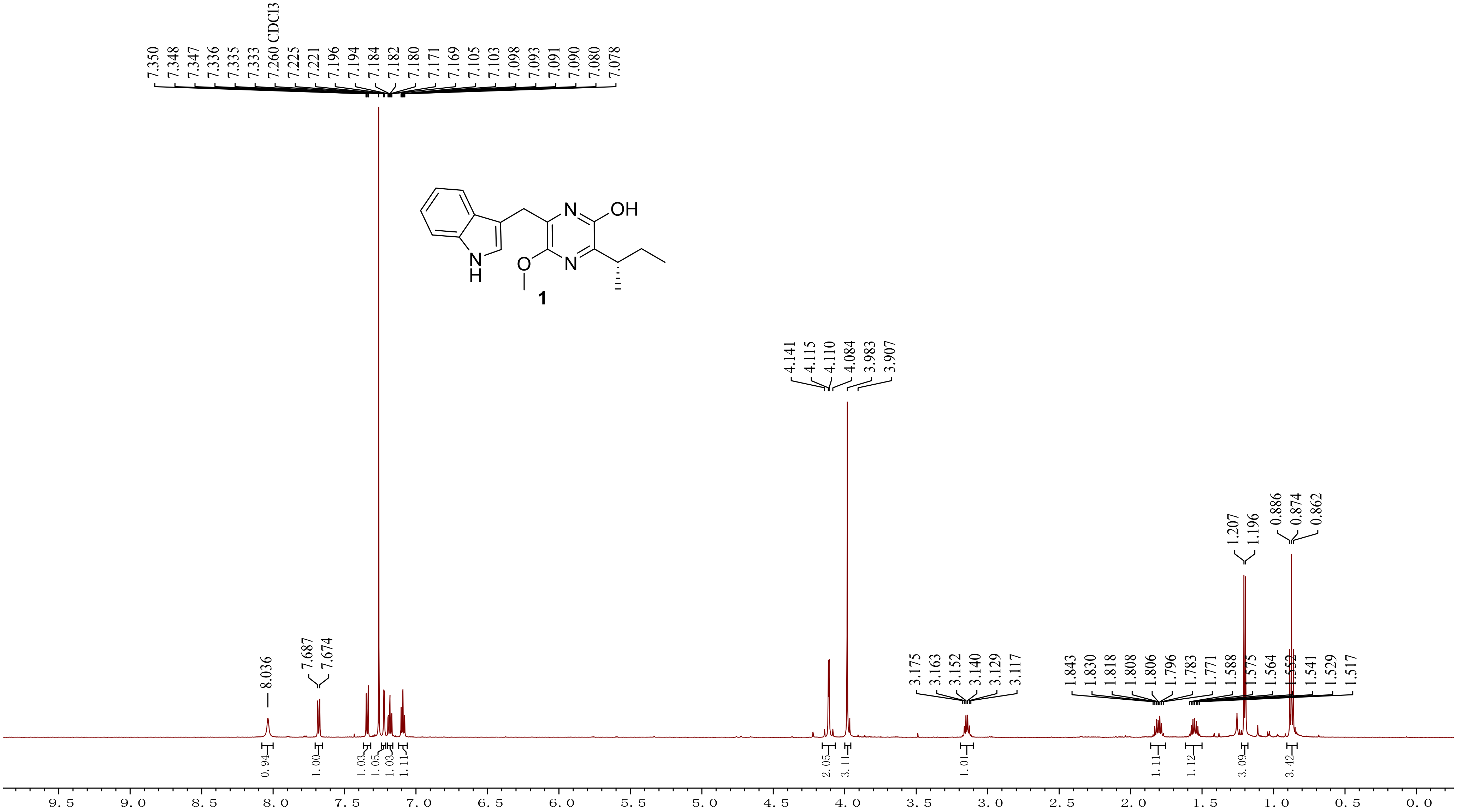

Figure S3. The ^{1}H NMR spectrum of trypilepyrazinol (**1**) in $CDCl_3$ (600 MHz).

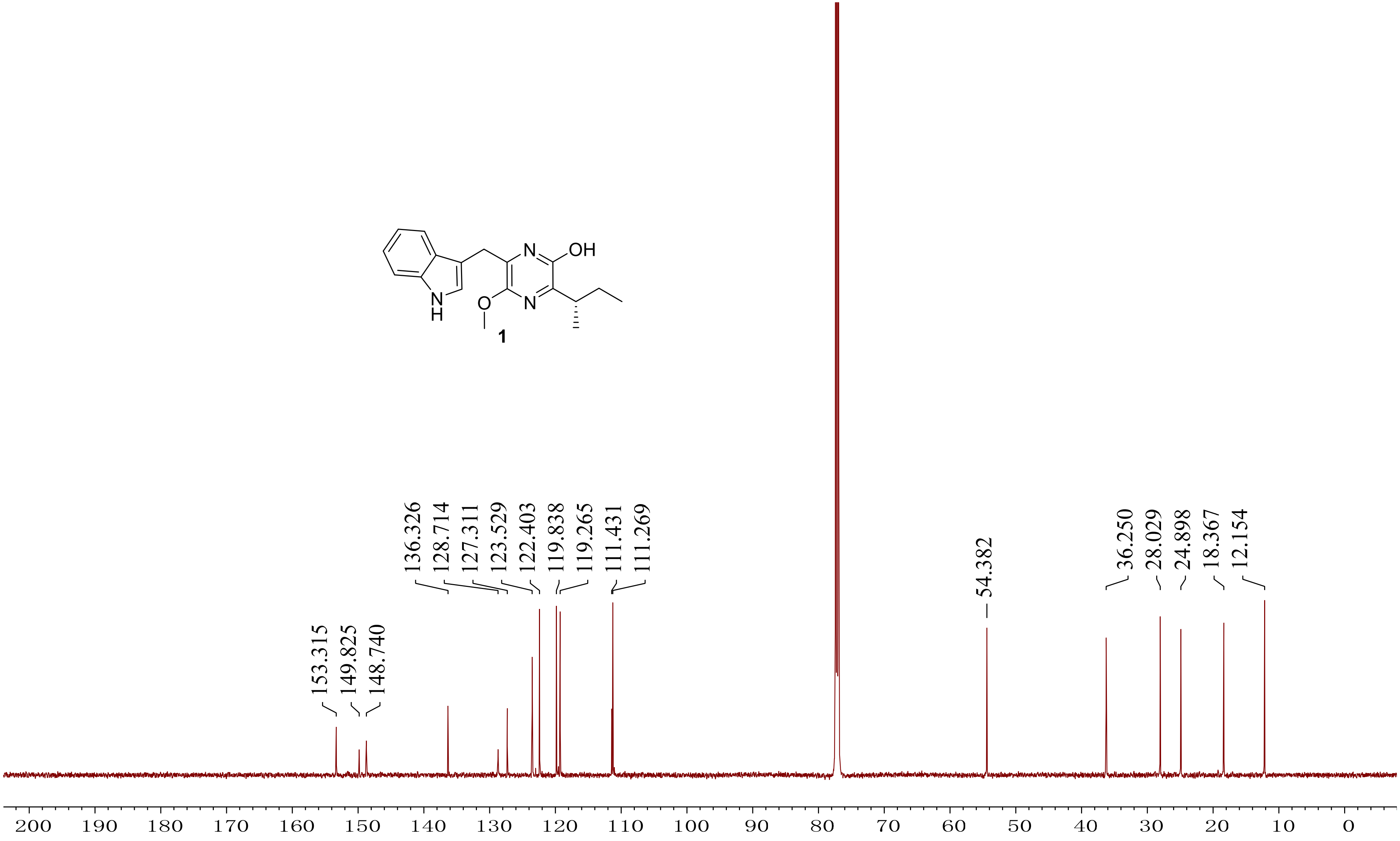

Figure S4. The ^{13}C NMR spectrum of trypilepyrazinol (**1**) in $CDCl_3$ (150 MHz)

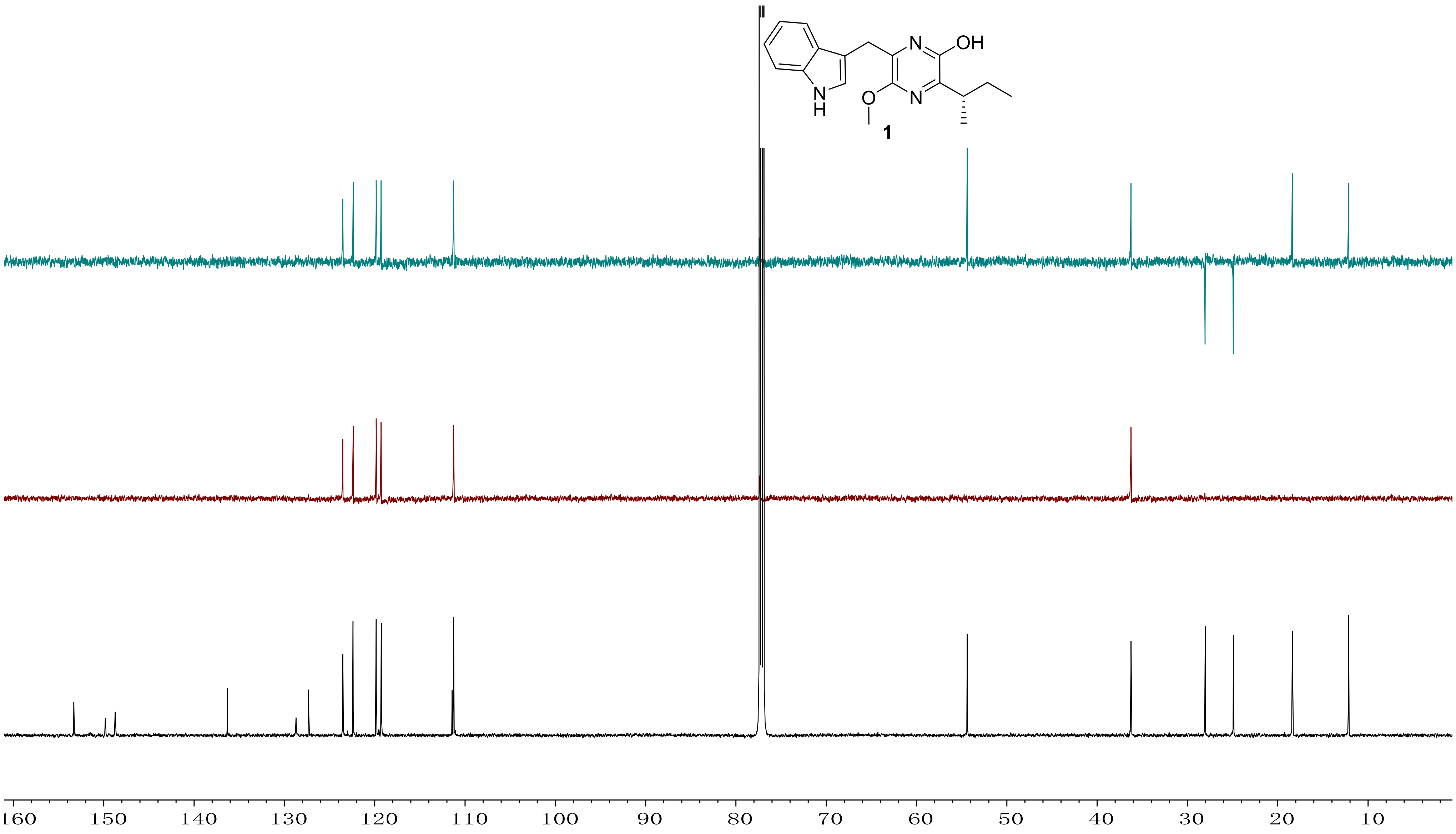

Figure S5. The DEPT spectrum of trypilepyrazinol (**1**) in $CDCl_3$ (150 MHz).

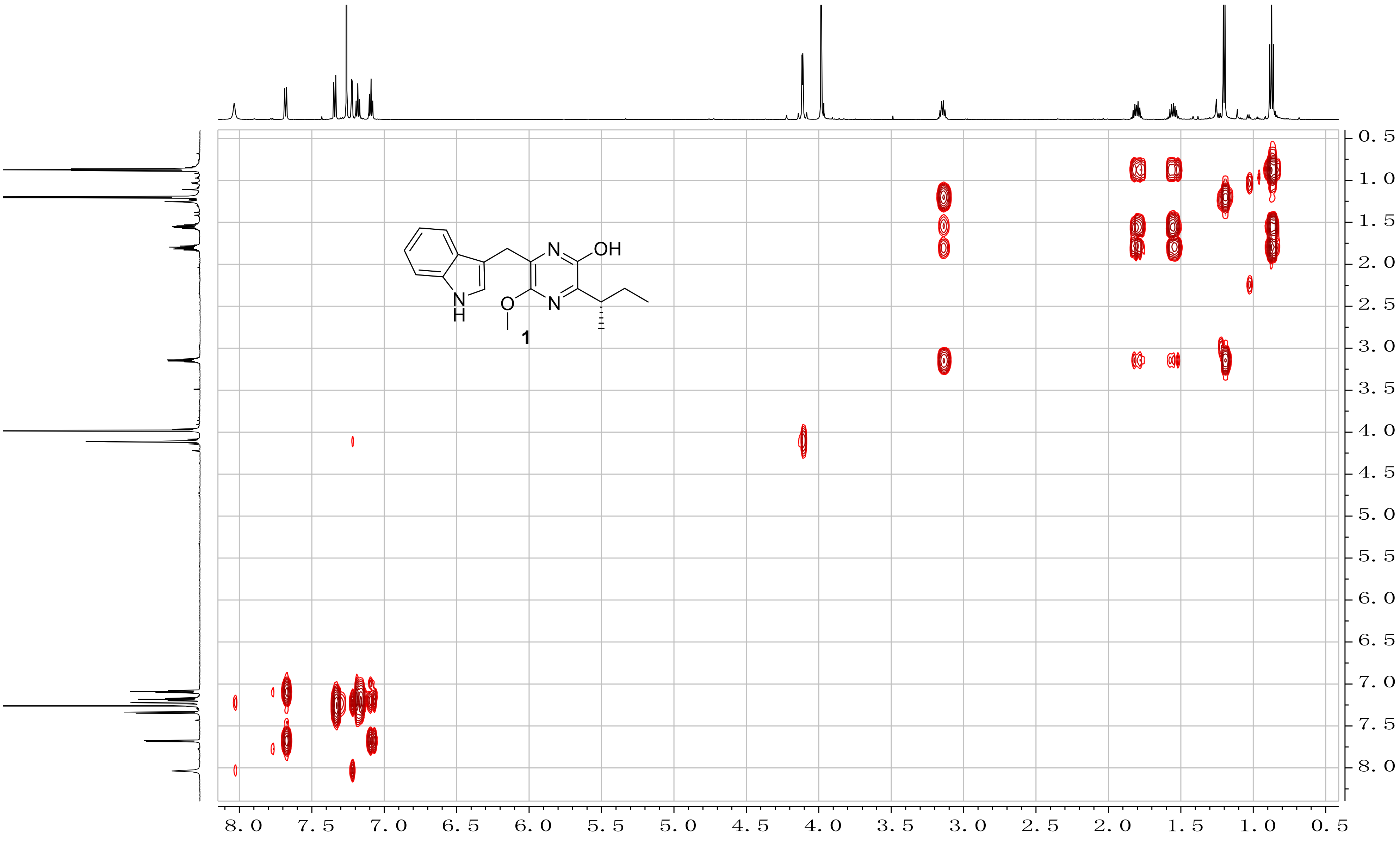

Figure S6. The ^{1}H-^{1}H COSY spectrum of trypilepyrazinol (**1**) in $CDCl_3$ (600 MHz).

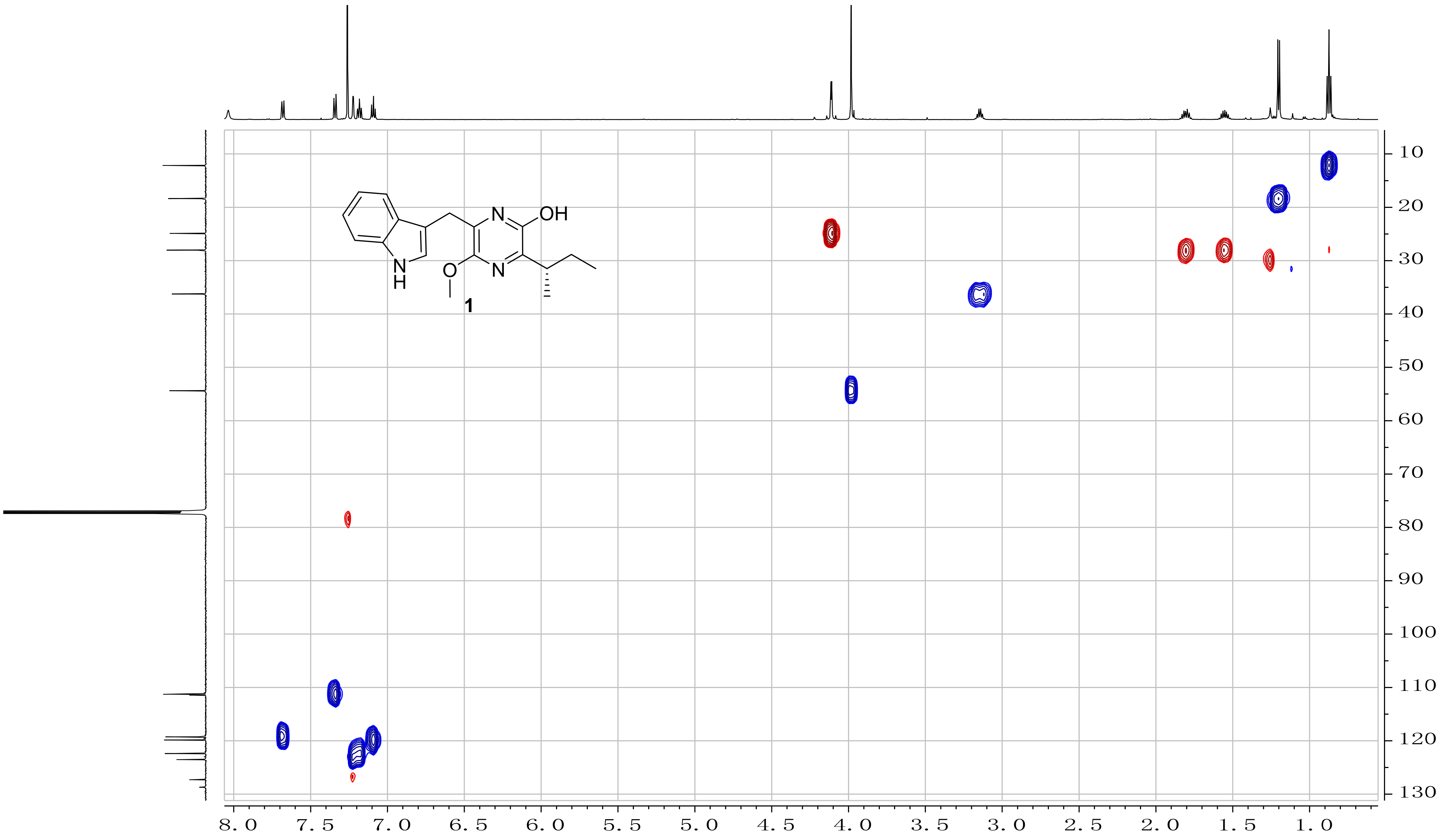

Figure S7. The HSQC spectrum of trypilepyrazinol (**1**) in $CDCl_3$ (600 MHz).

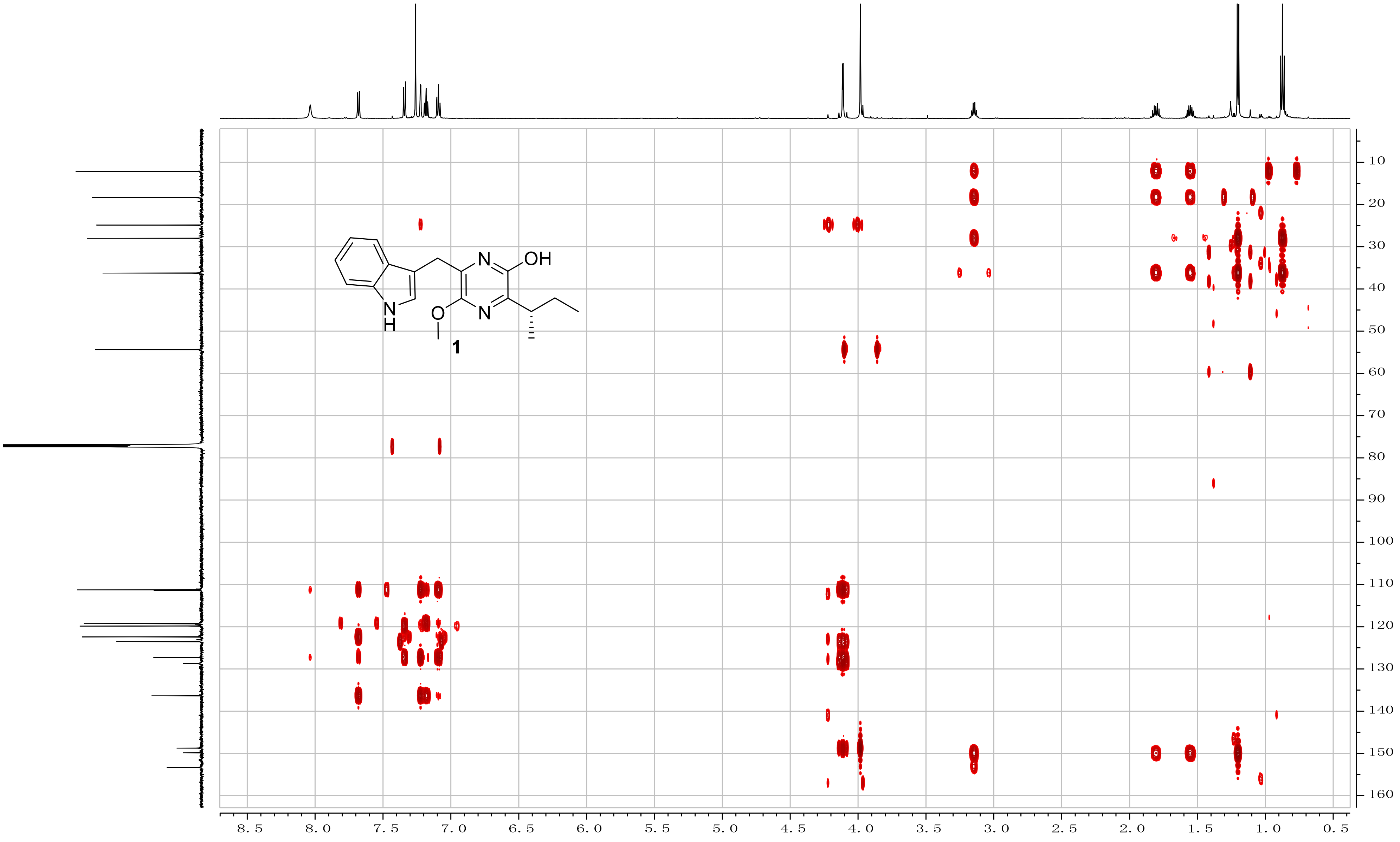

Figure S8. The HMBC spectrum of trypilepyrazinol (**1**) in $CDCl_3$ (600 MHz).

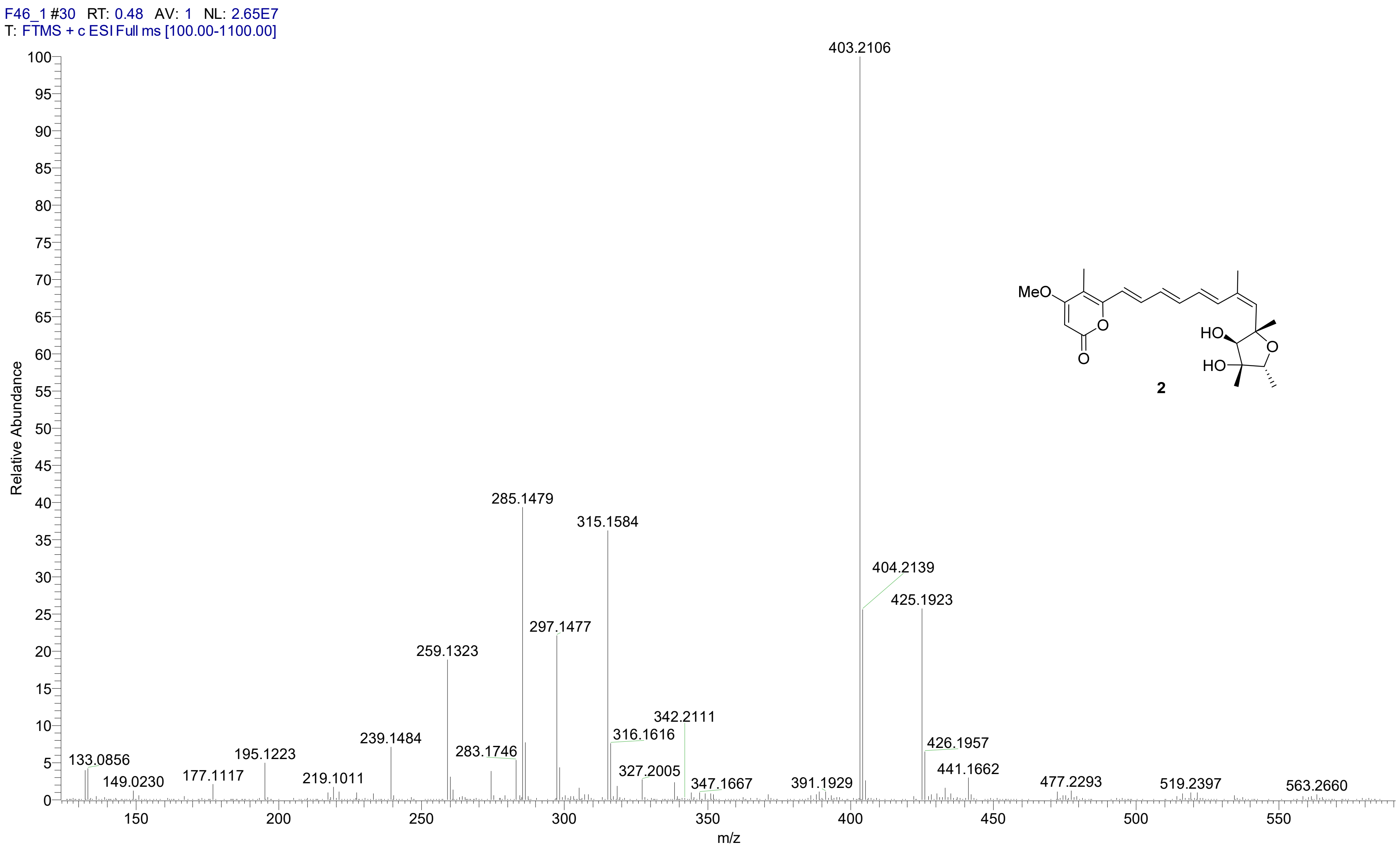

Figure S9. The (+)-HRESIMS spectrum of (+)-neocitreoviridin (**2**).

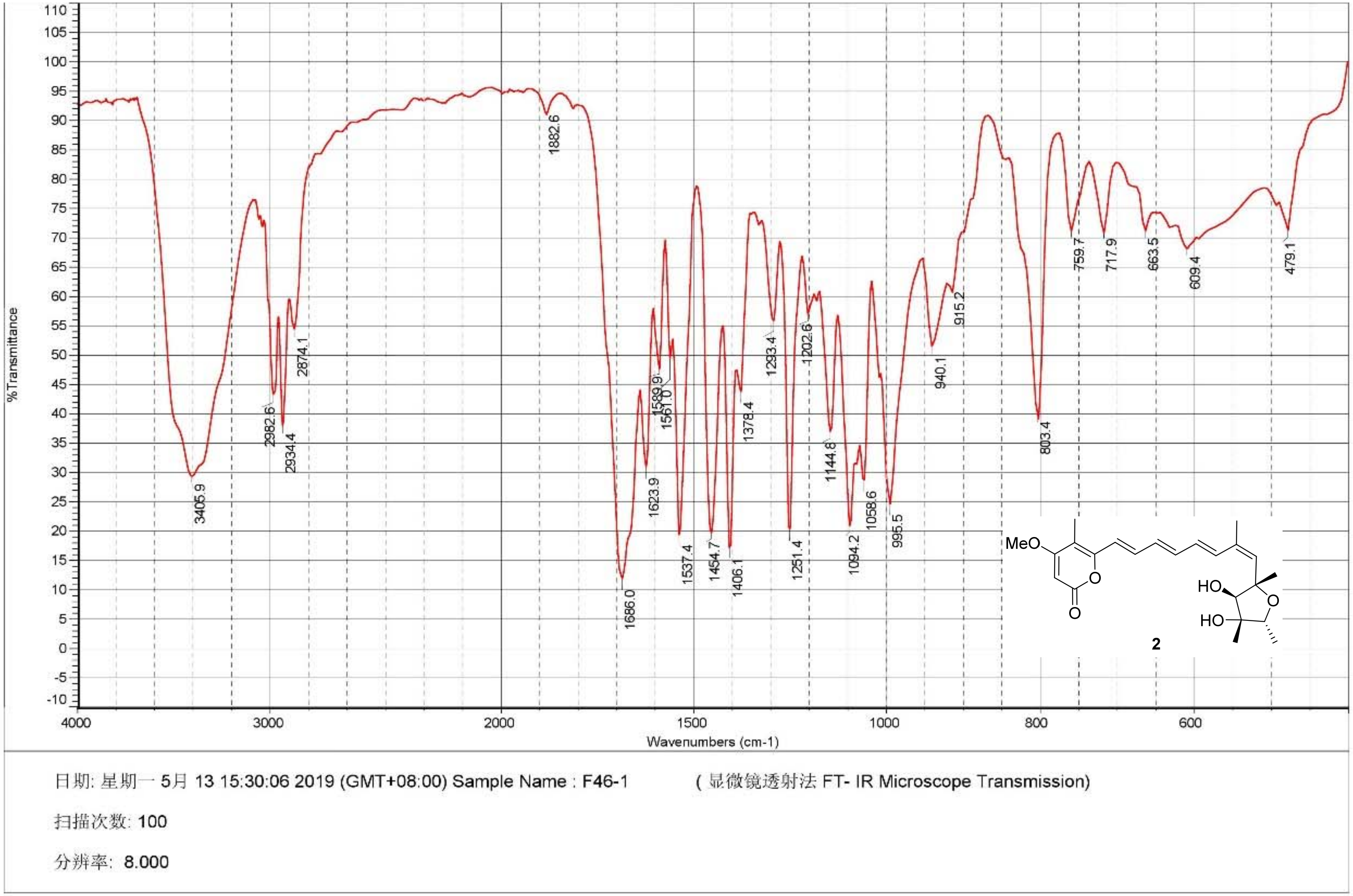

Figure S10. The IR spectrum of (+)-neocitreoviridin (**2**).

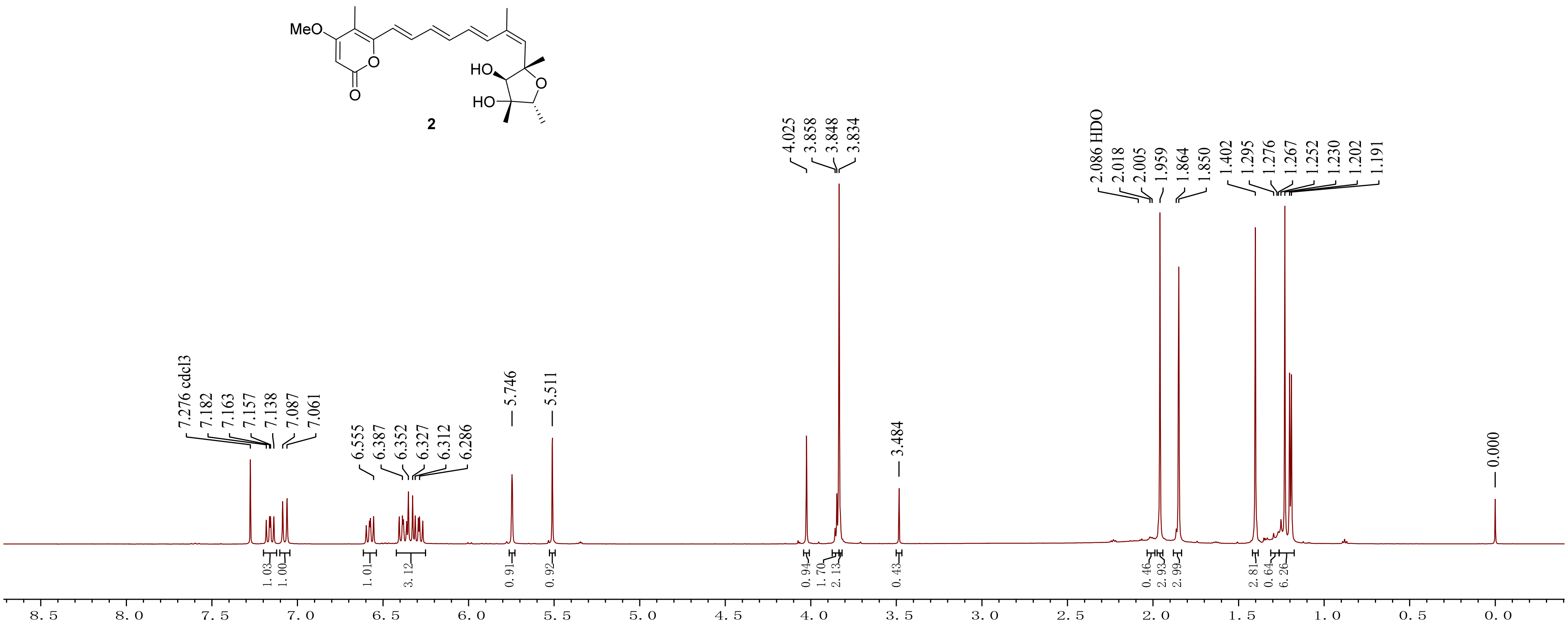

Figure S11. The ^{1}H NMR spectrum of (+)-neocitreoviridin (**2**) in $CDCl_3$ (600 MHz).

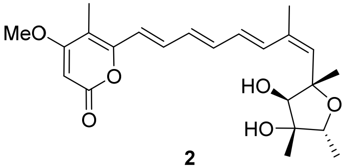

2

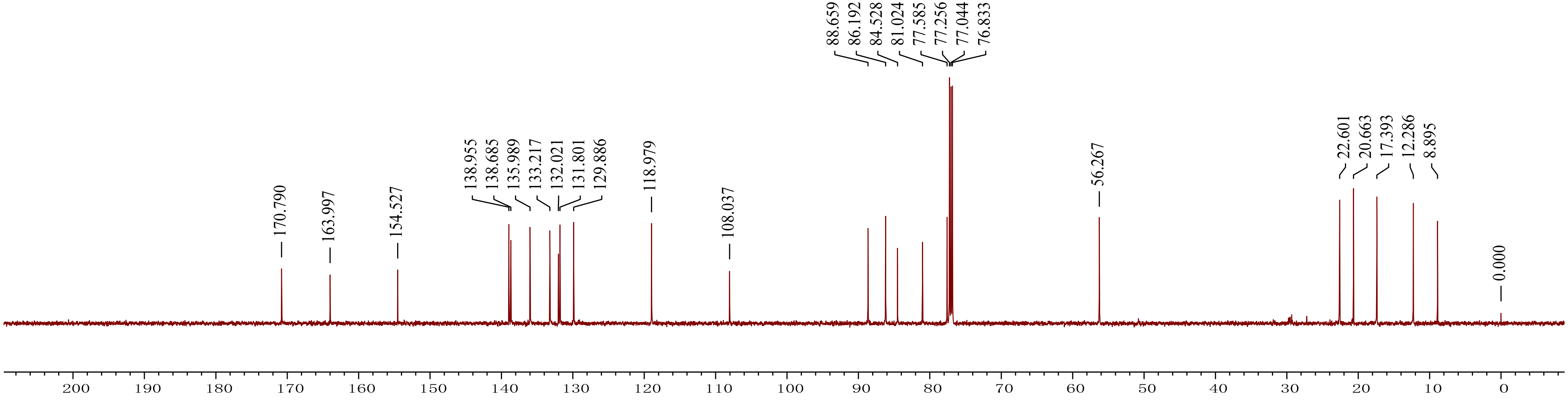

Figure S12. The ^{13}C NMR spectrum of (+)-neocitreoviridin (**2**) in $CDCl_3$ (150 MHz).

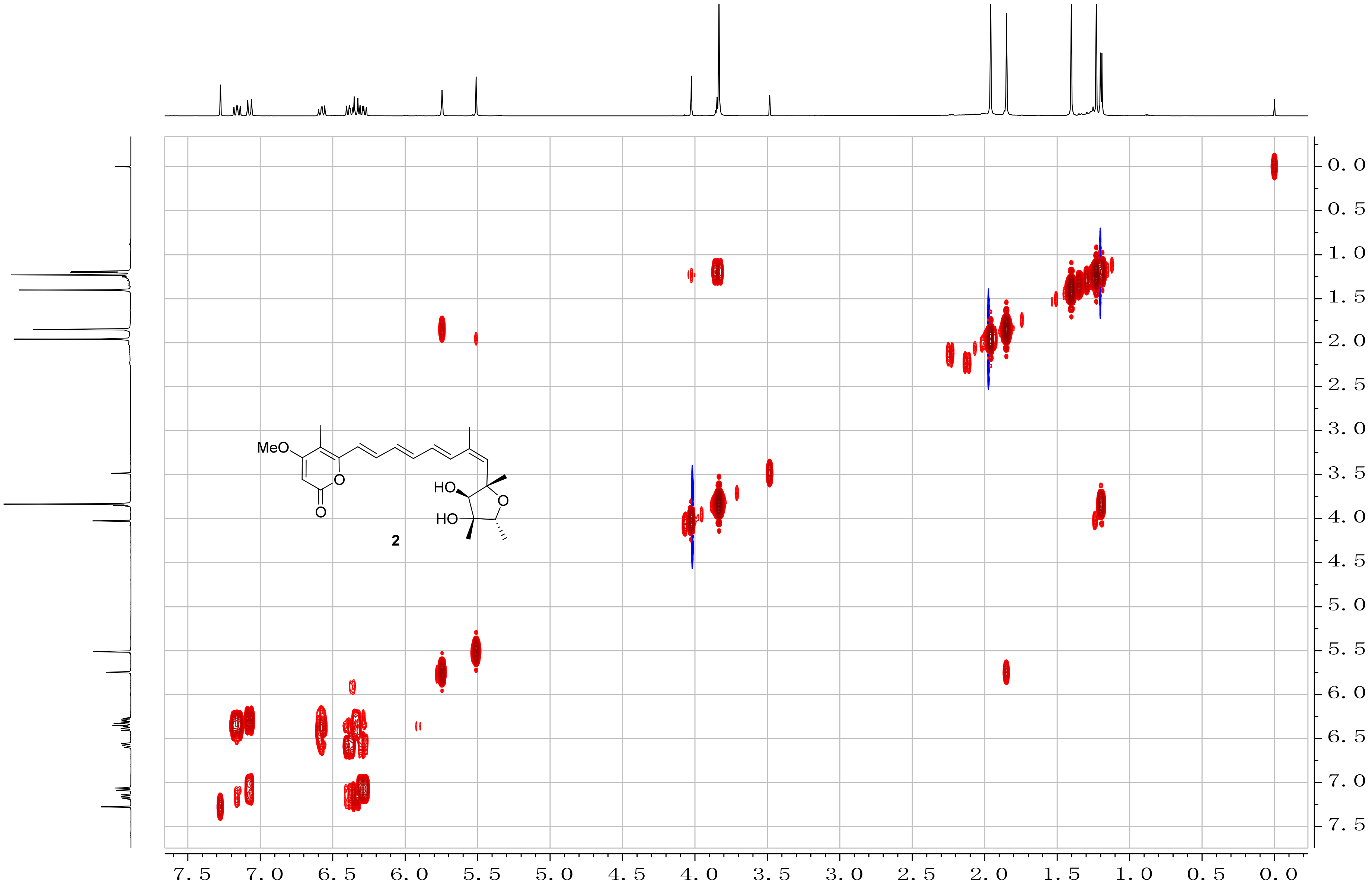

Figure S13. The ^{1}H-^{1}H COSY spectrum of (+)-neocitreoviridin (**2**) in $CDCl_3$ (600 MHz)

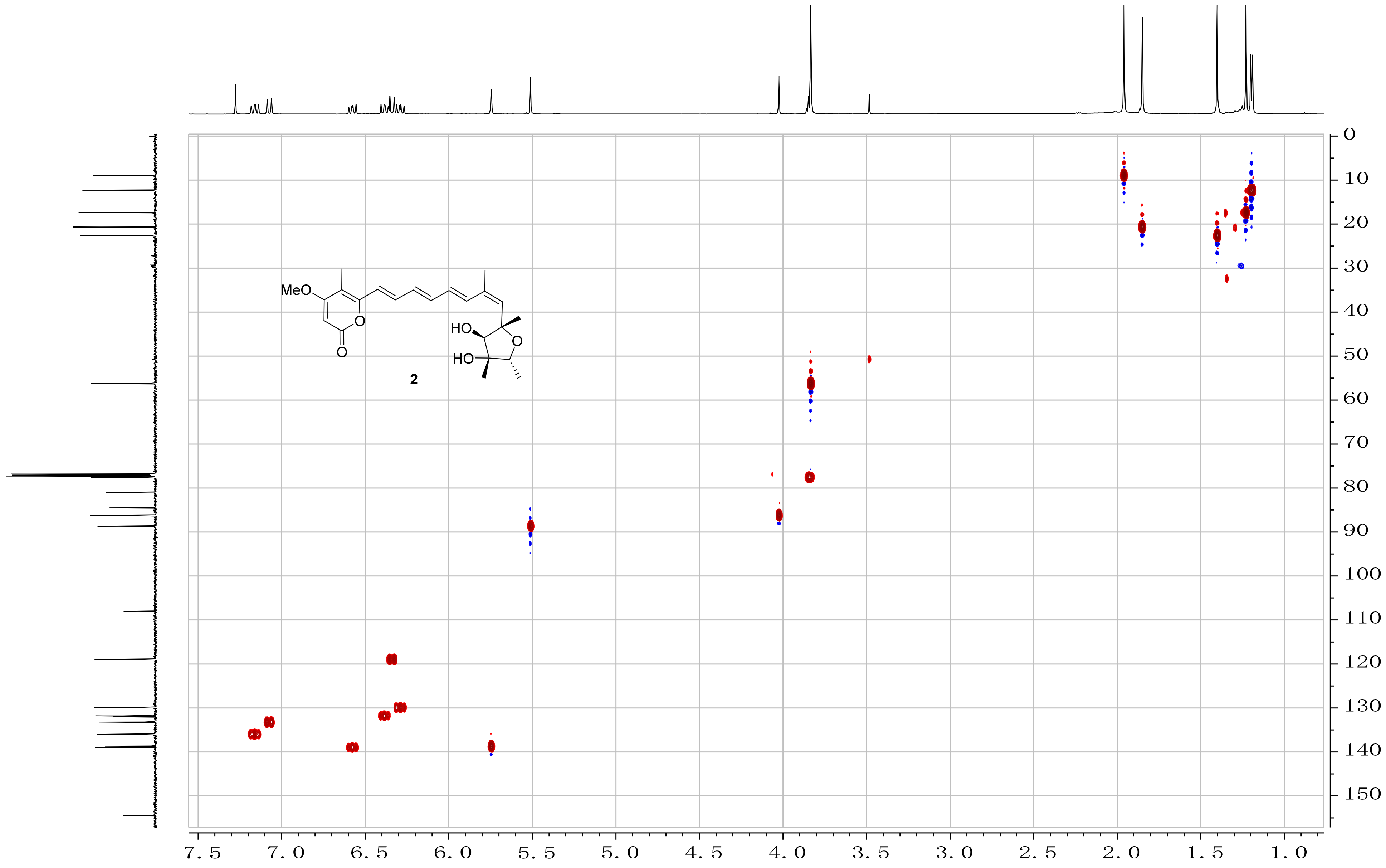

Figure S14. The HSQC spectrum of (+)-neocitreoviridin (**2**) in $CDCl_3$ (600 MHz).

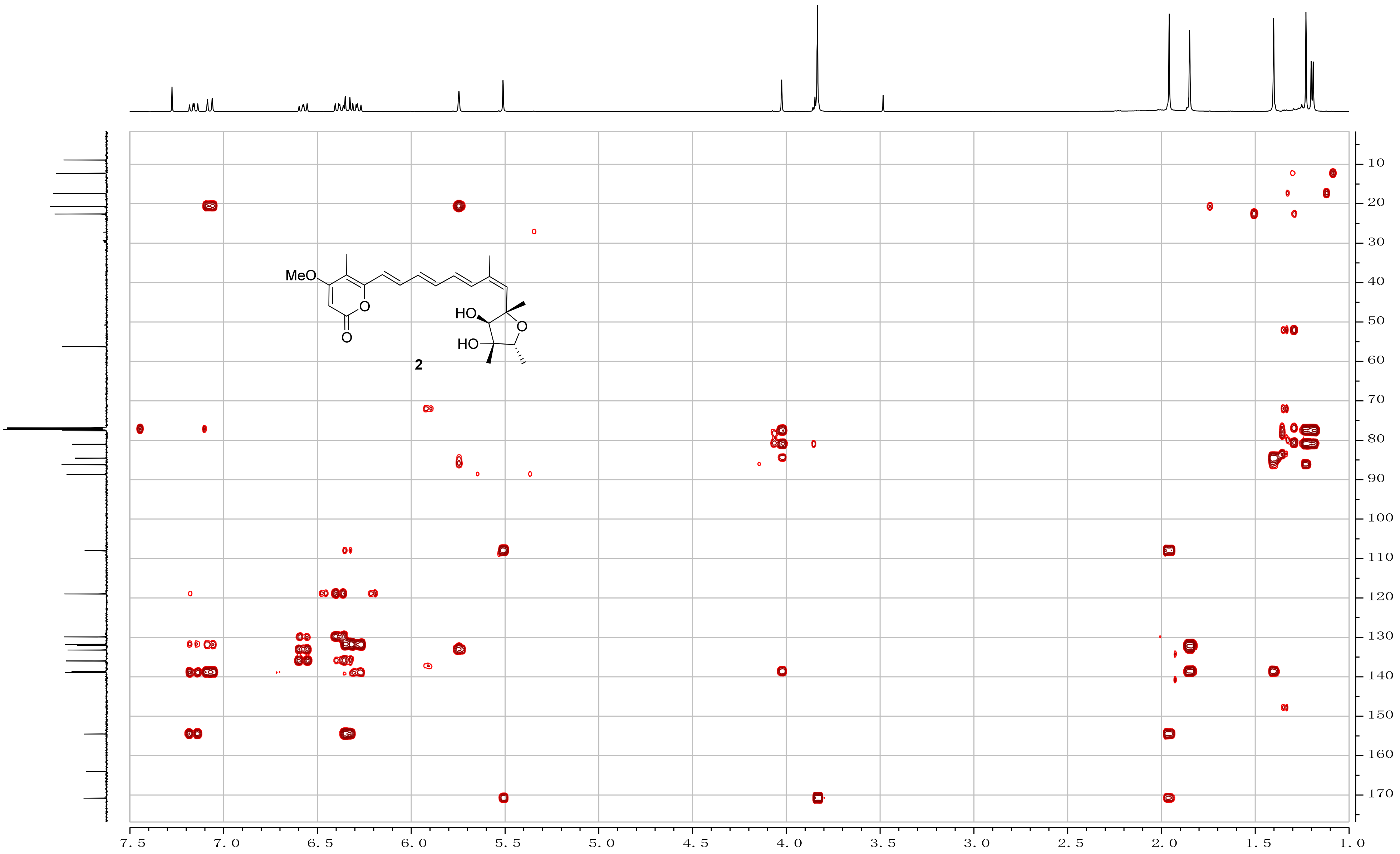

Figure S15. The HMBC spectrum of (+)-neocitreoviridin (**2**) in $CDCl_3$ (600 MHz).

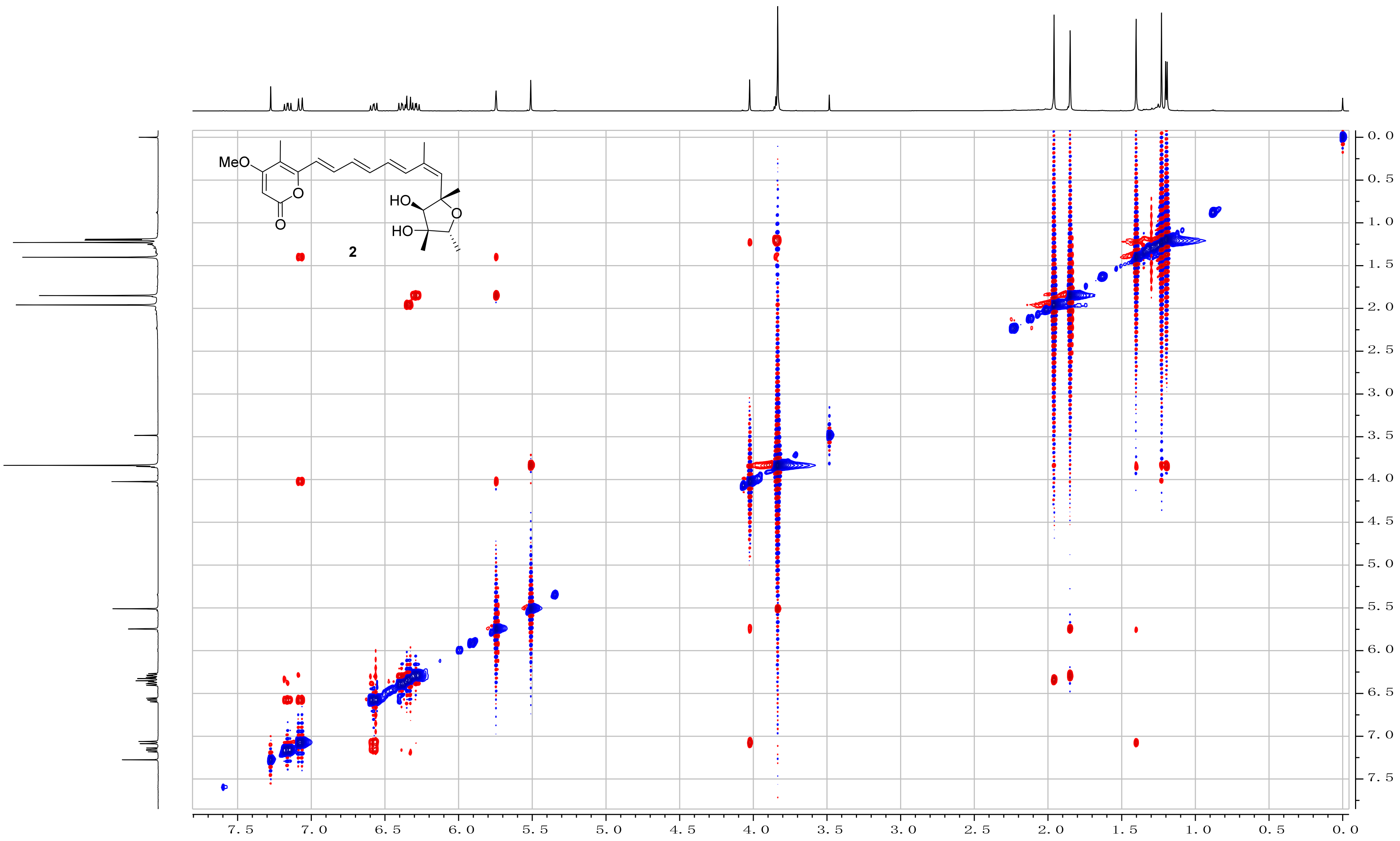

Figure S16. The ROESY spectrum of (+)-neocitreoviridin (**2**) in $CDCl_3$ (600 MHz).

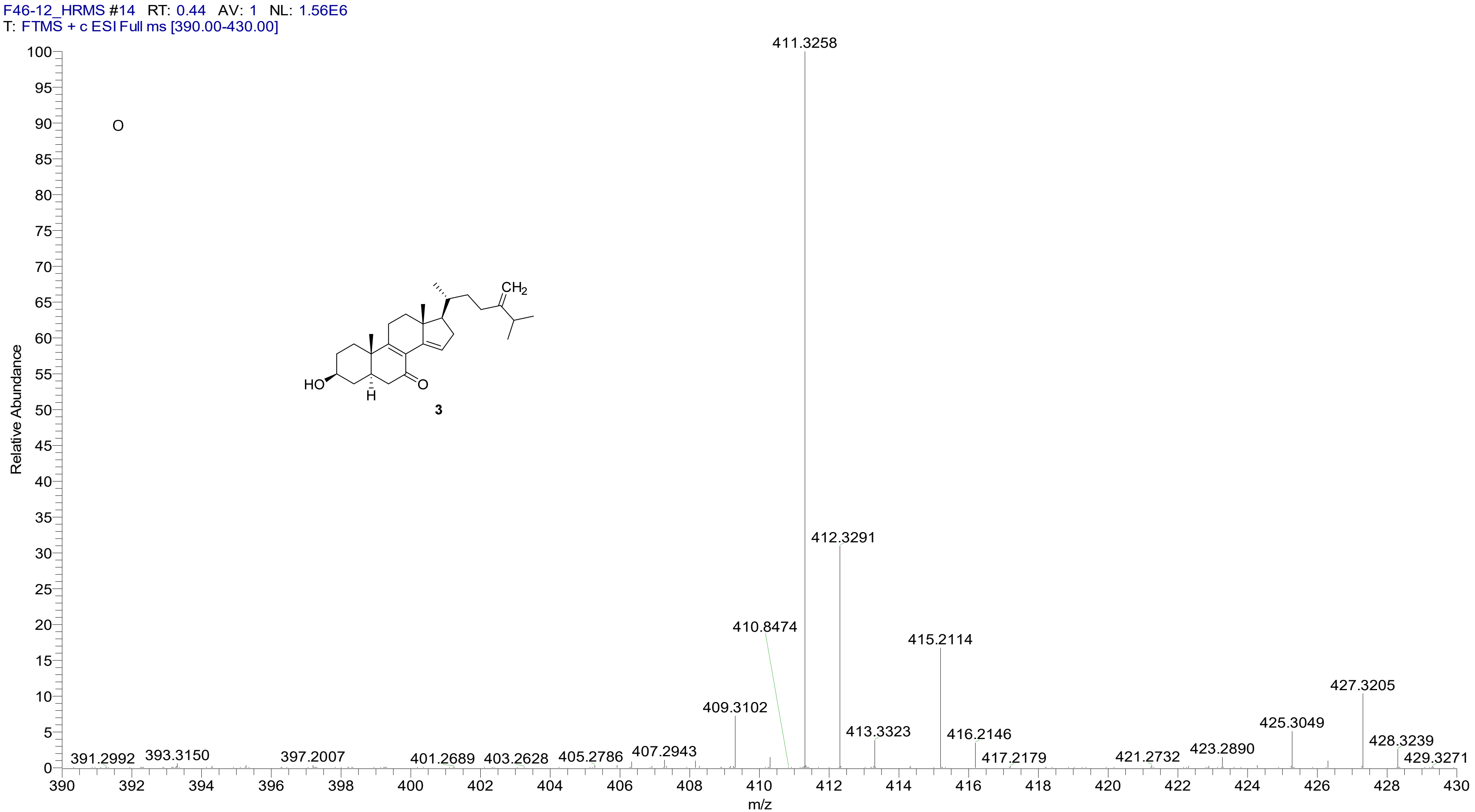

Figure S17. The (+)-HRESIMS spectrum of 3β-hydroxyergosta-8,14,24(28)-trien-7-one (**3**).

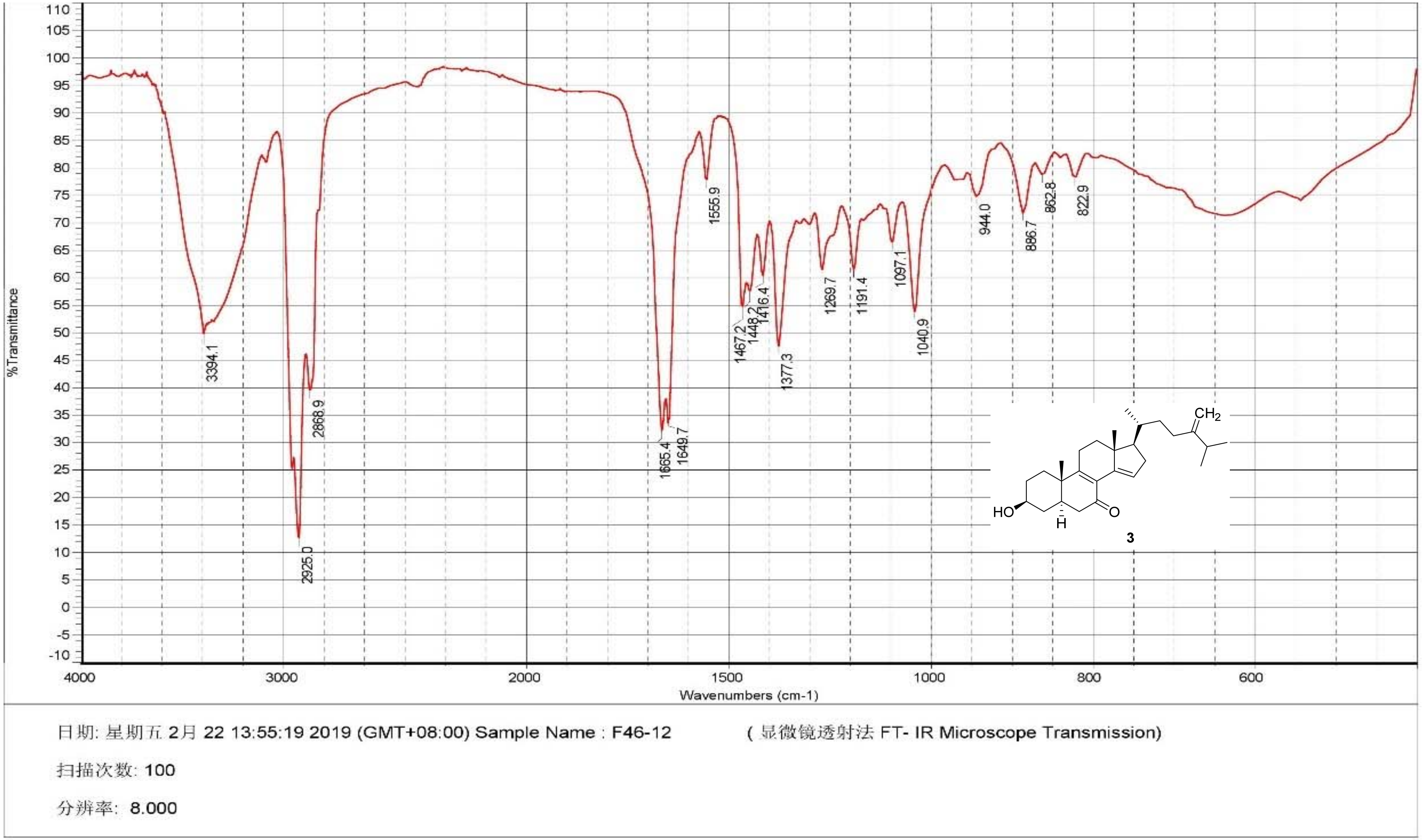

Figure S18. The IR spectrum of 3*β*-hydroxyergosta-8,14,24(28)-trien-7-one (**3**).

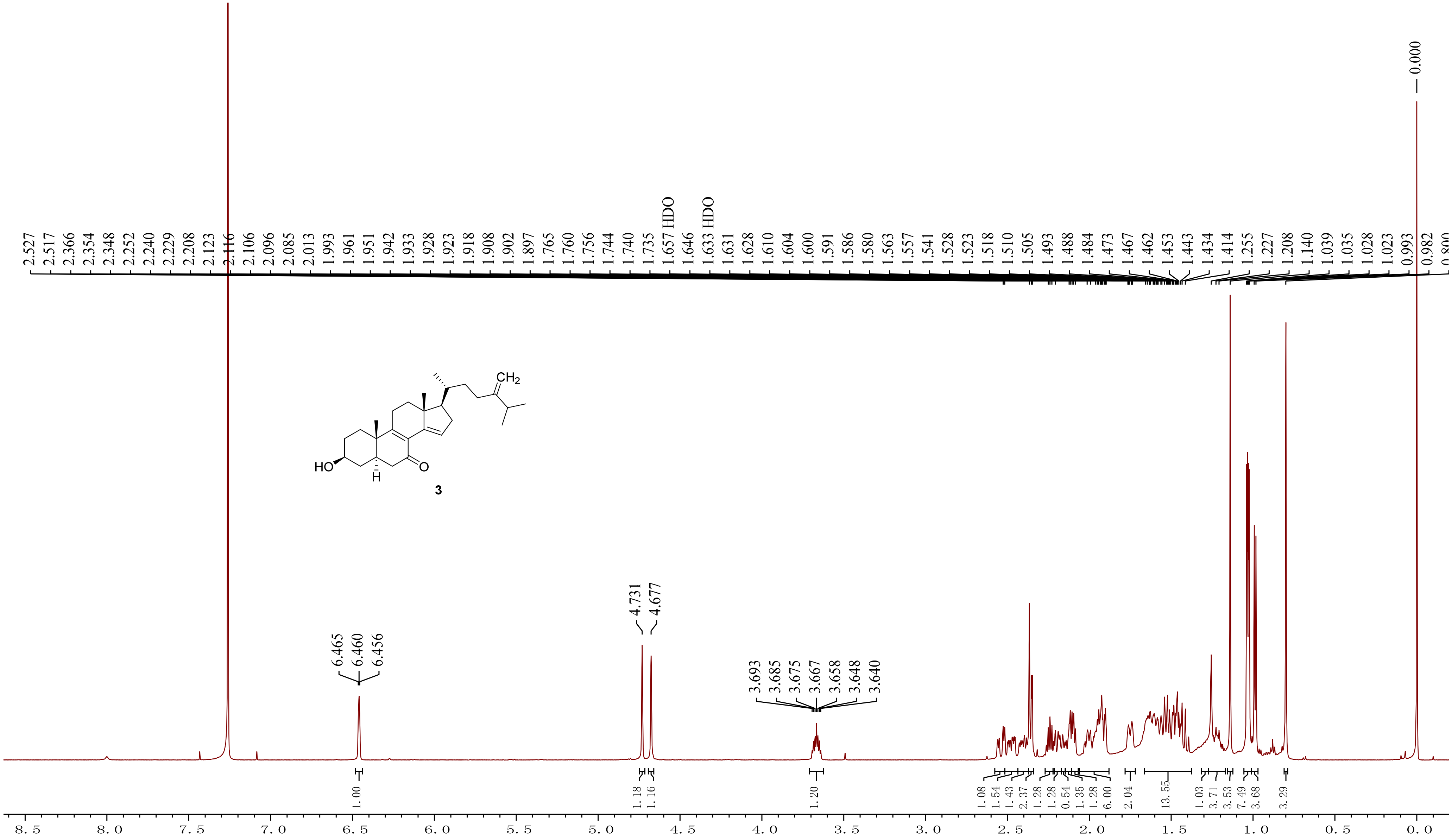

Figure S19. The ^{1}H NMR spectrum of 3*β*-hydroxyergosta-8,14,24(28)-trien-7-one (**3**) in $CDCl_3$ (600 MHz).

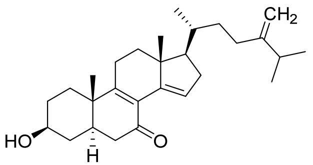

3

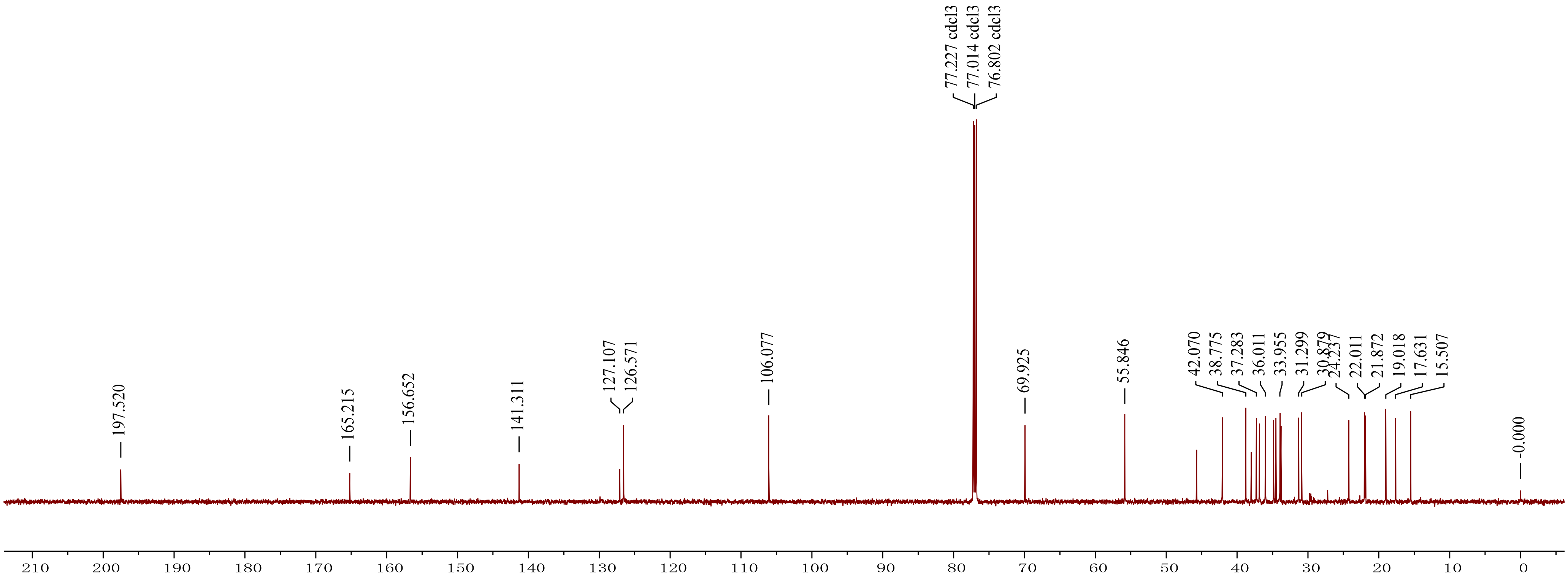

Figure S20. The ^{13}C NMR spectrum of 3*β*-hydroxyergosta-8,14,24(28)-trien-7-one (**3**) in $CDCl_3$ (150 MHz).

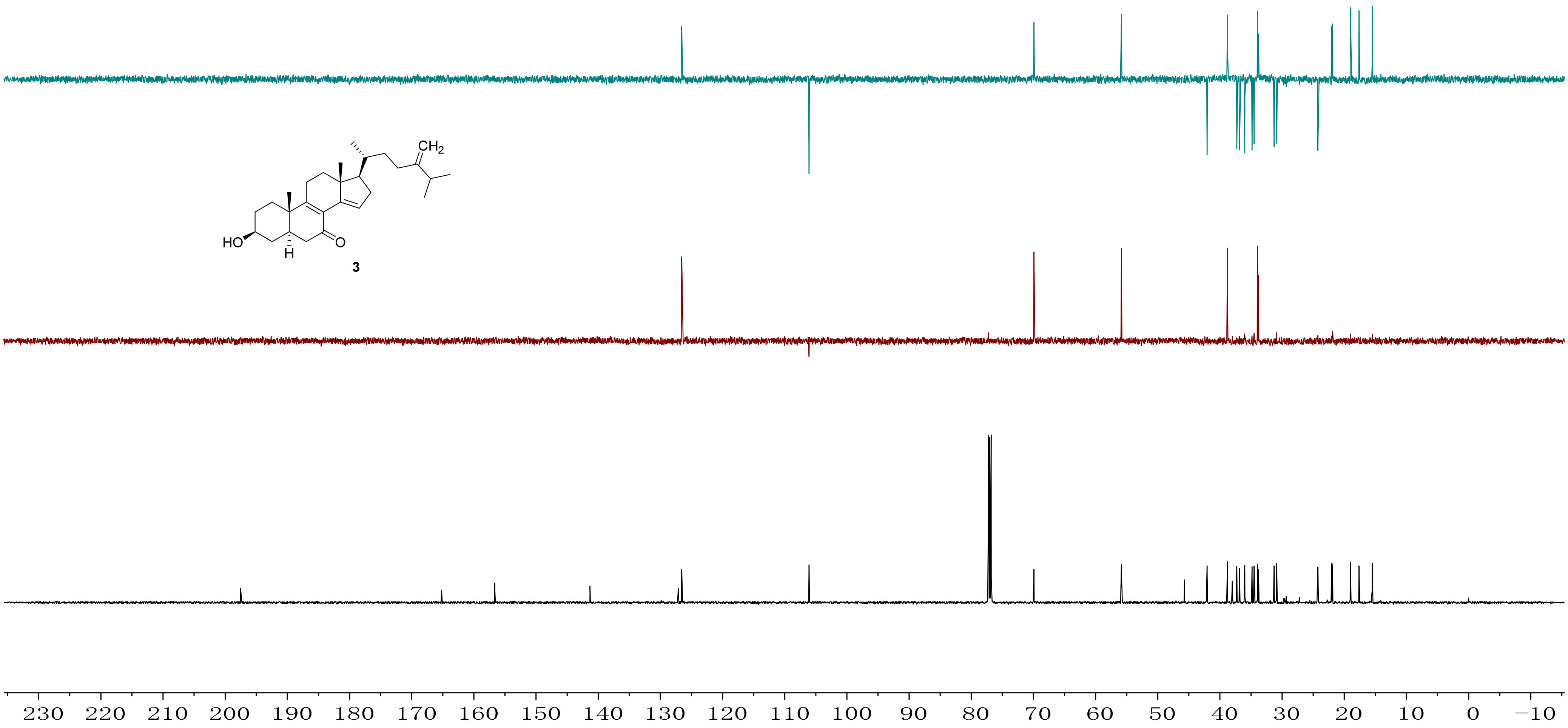

Figure S21. The DEPT spectrum of 3*β*-hydroxyergosta-8,14,24(28)-trien-7-one (**3**) in $CDCl_3$ (150 MHz).

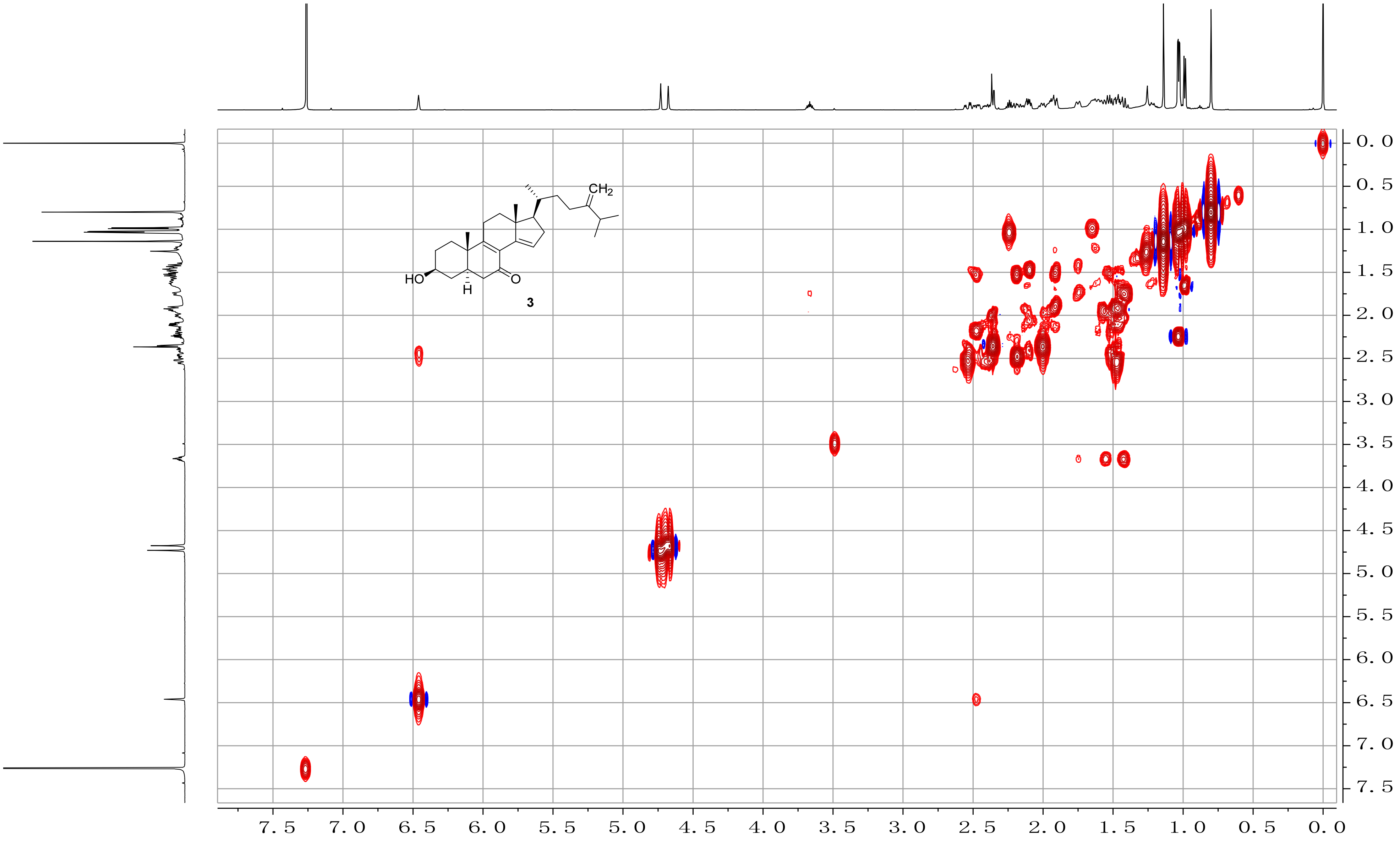

Figure S22. The ^{1}H-^{1}H COSY spectrum of 3β-hydroxyergosta-8,14,24(28)-trien-7-one (**3**) in $CDCl_3$ (600 MHz).

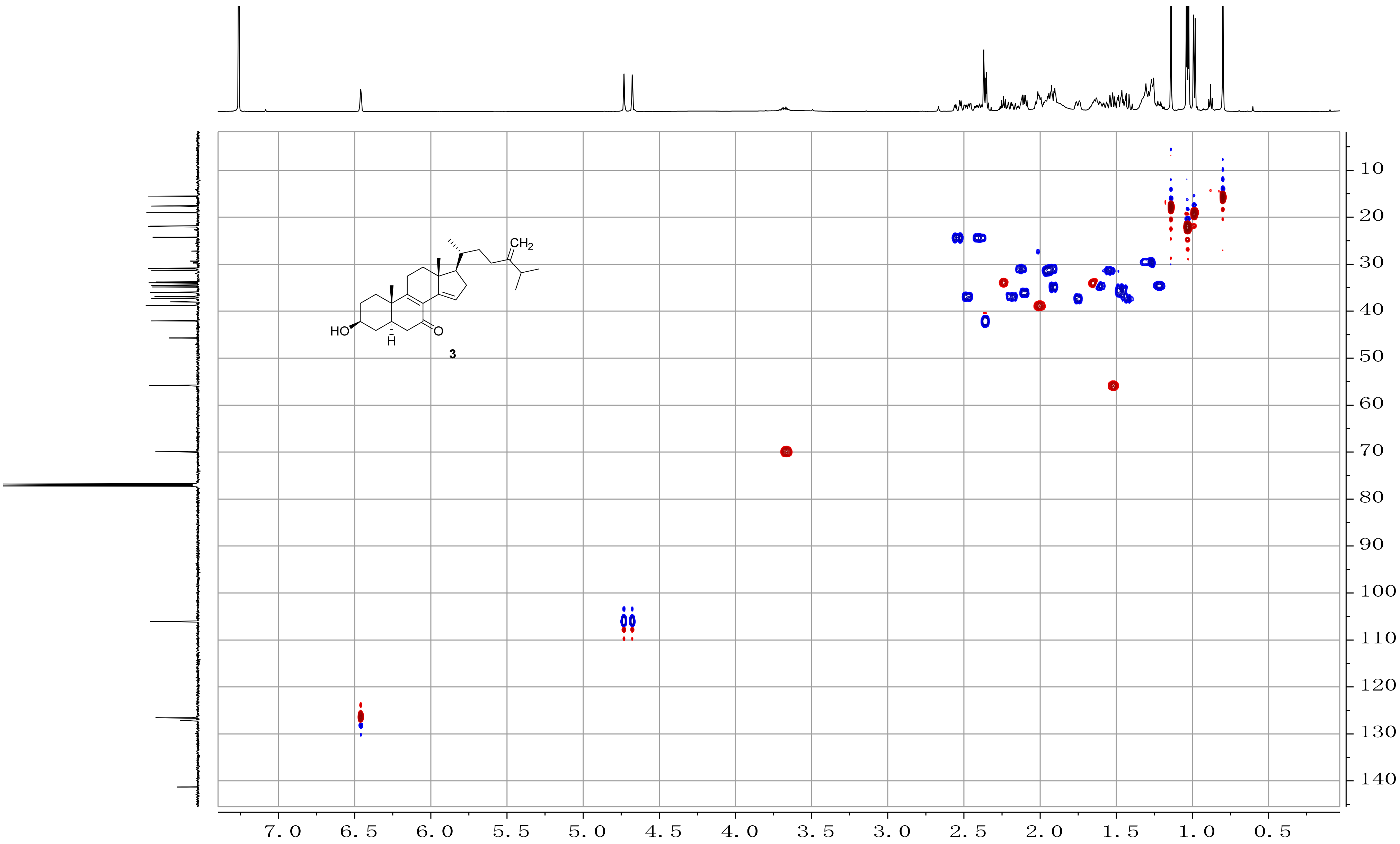

Figure S23. The HSQC spectrum of 3*β*-hydroxyergosta-8,14,24(28)-trien-7-one (**3**) in $CDCl_3$ (600 MHz).

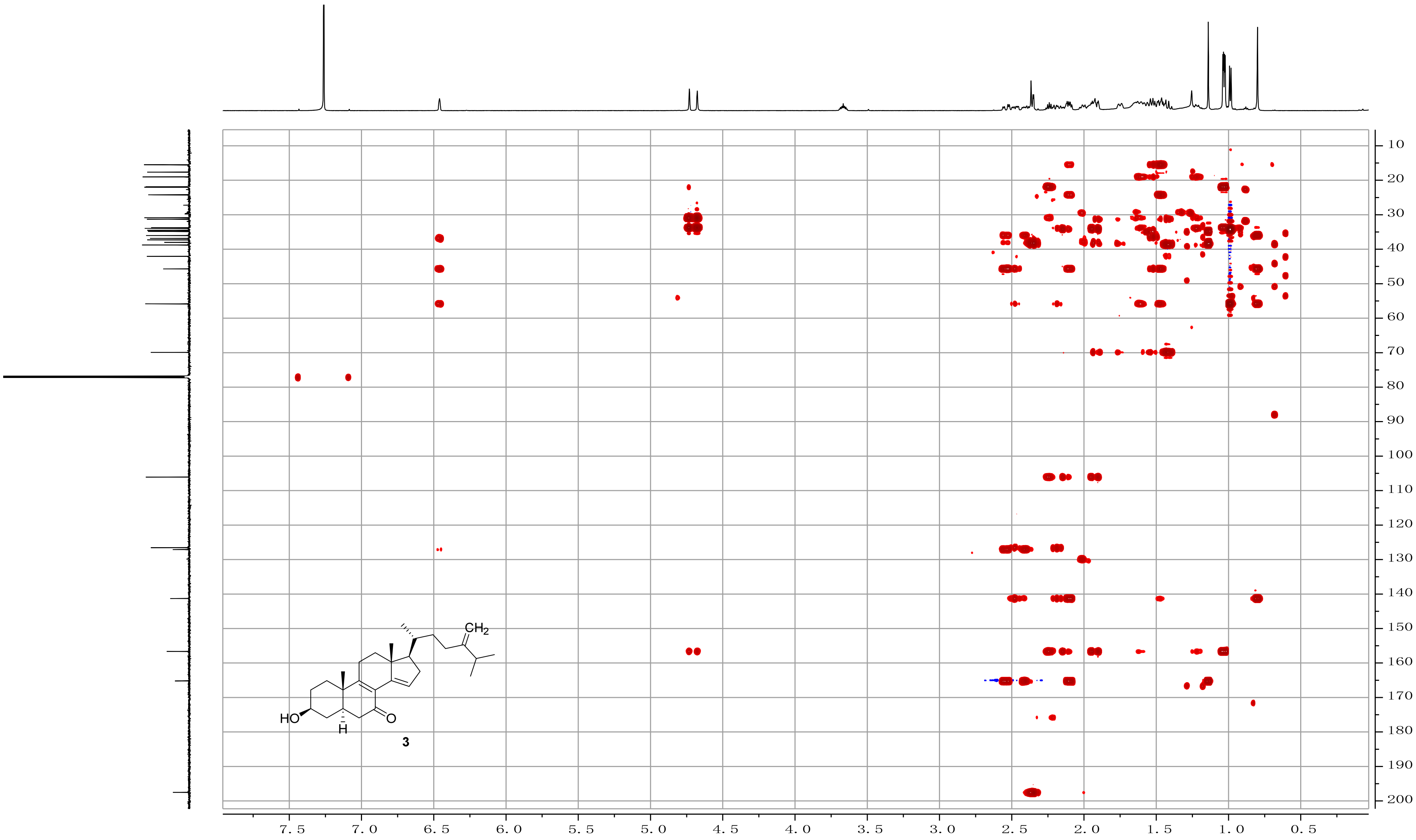

Figure S24. The HMBC spectrum of 3β-hydroxyergosta-8,14,24(28)-trien-7-one (**3**) in $CDCl_3$ (600 MHz).

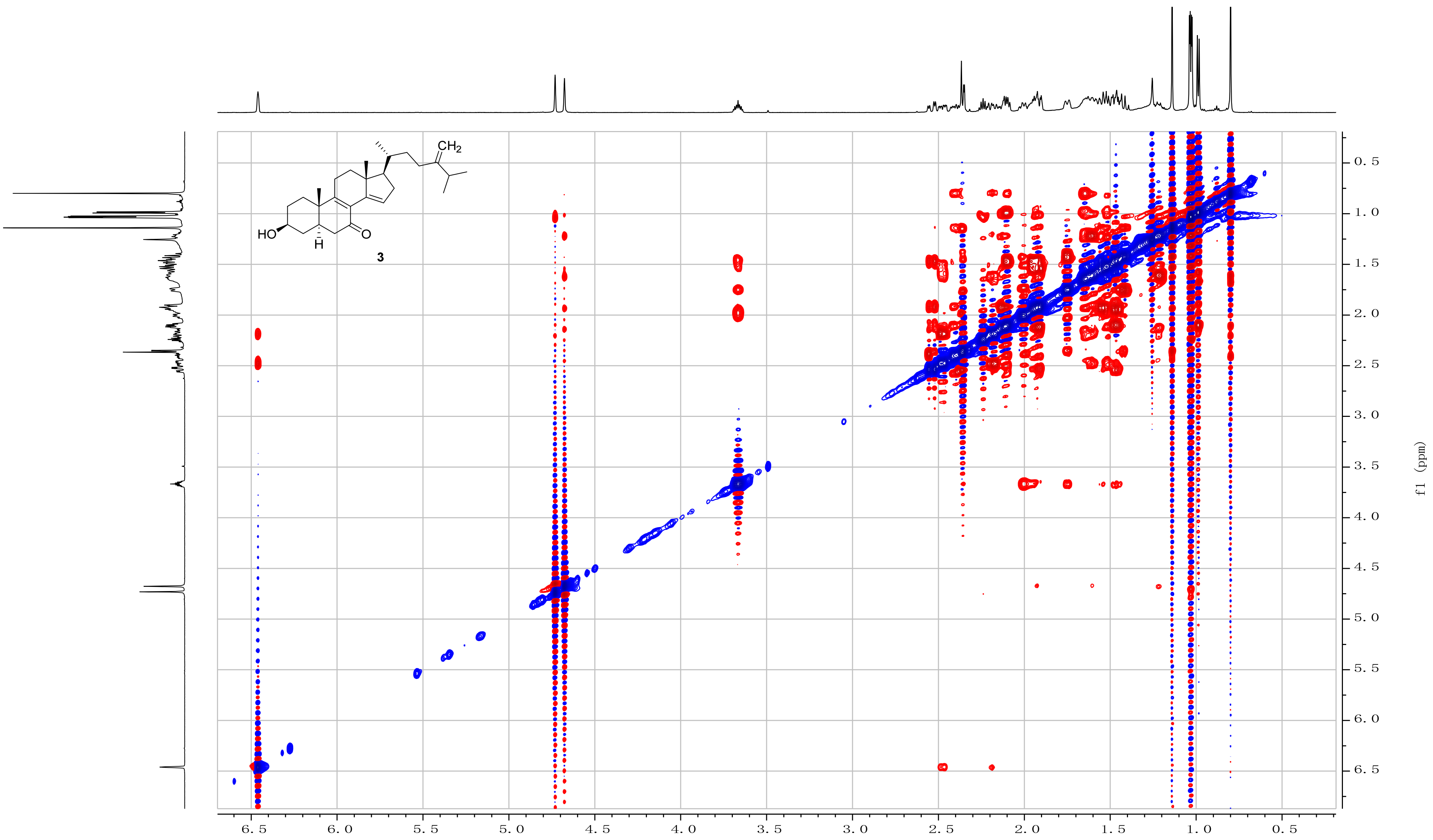

Figure S25. The ROESY spectrum of 3β-hydroxyergosta-8,14,24(28)-trien-7-one (**3**) in $CDCl_3$ (600 MHz)

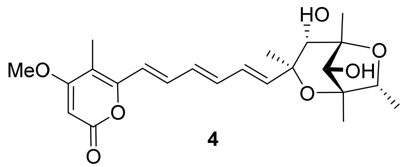

4

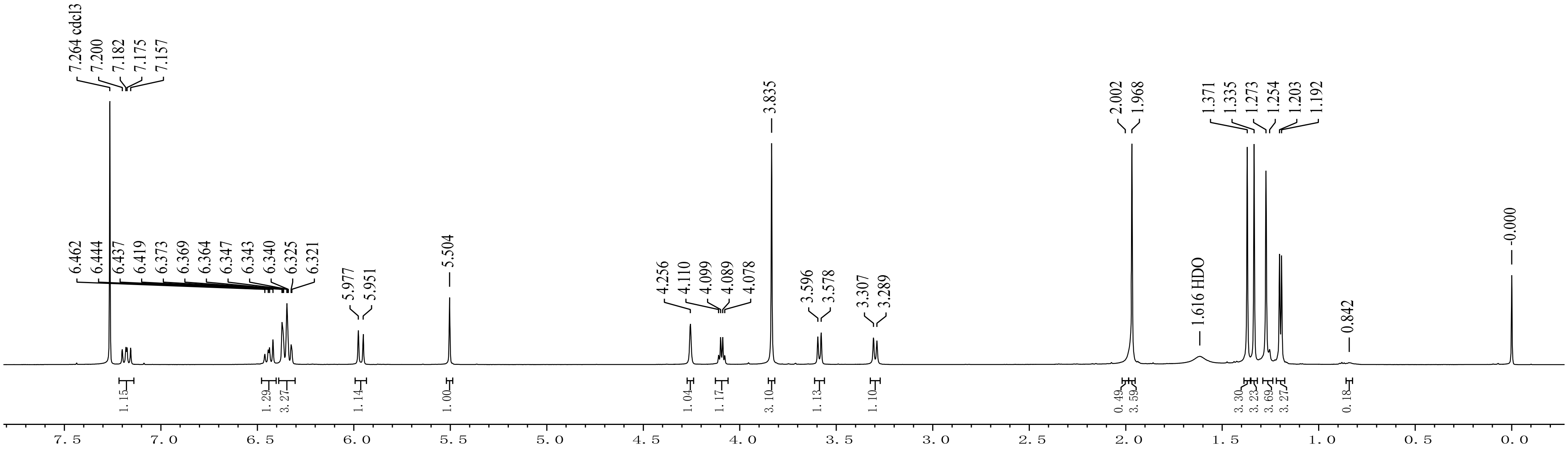

Figure S26. The ^{1}H NMR spectrum of epiisocitreoviridinol (**4**) in $CDCl_3$ (600 MHz).

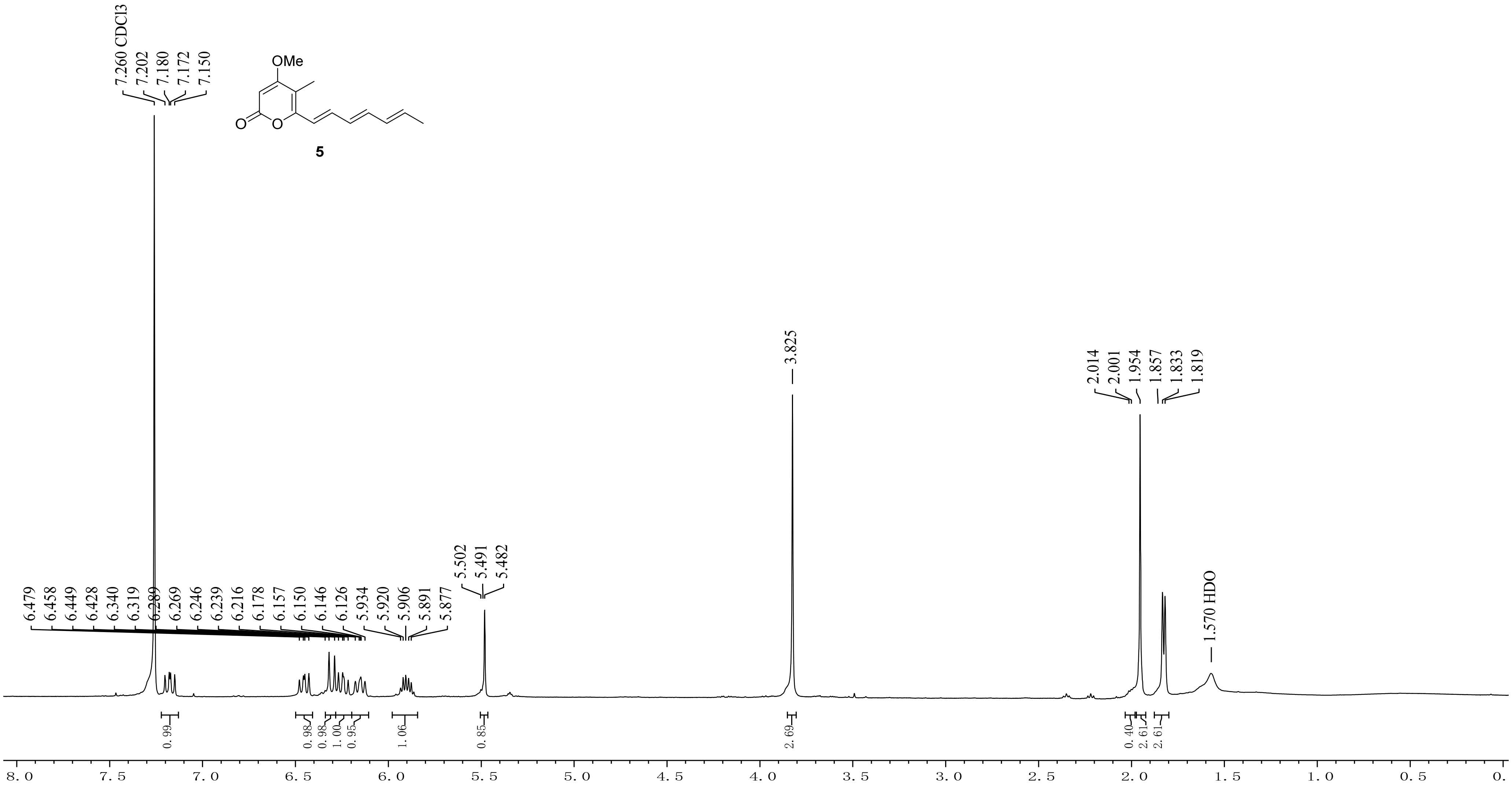

Figure S27. The ^{1}H NMR spectrum of citreoviripyrone B (**5**) in $CDCl_3$ (600 MHz).

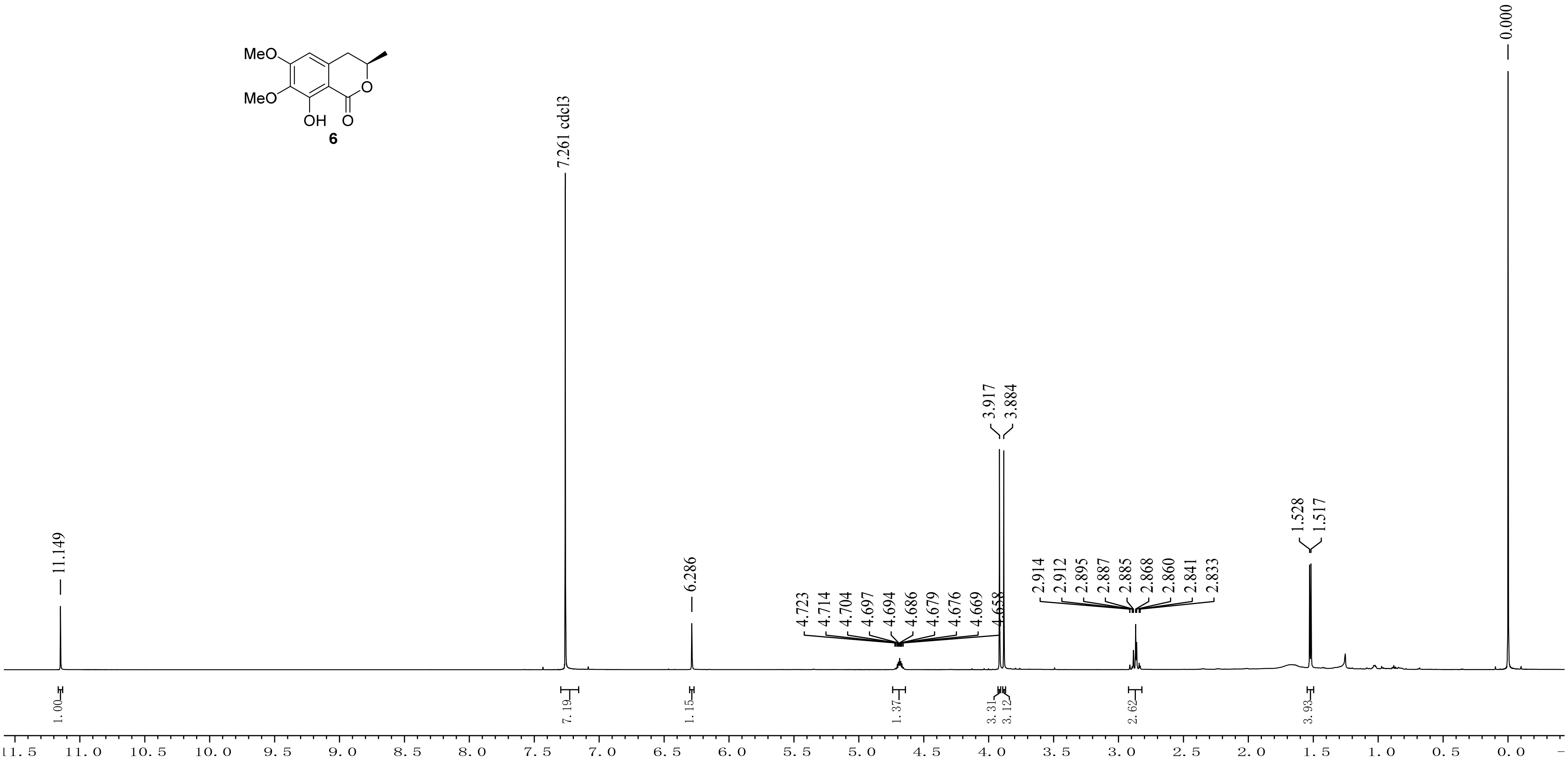

Figure S28. The ^{1}H NMR spectrum of kigelin (**6**) in $CDCl_3$ (600 MHz).

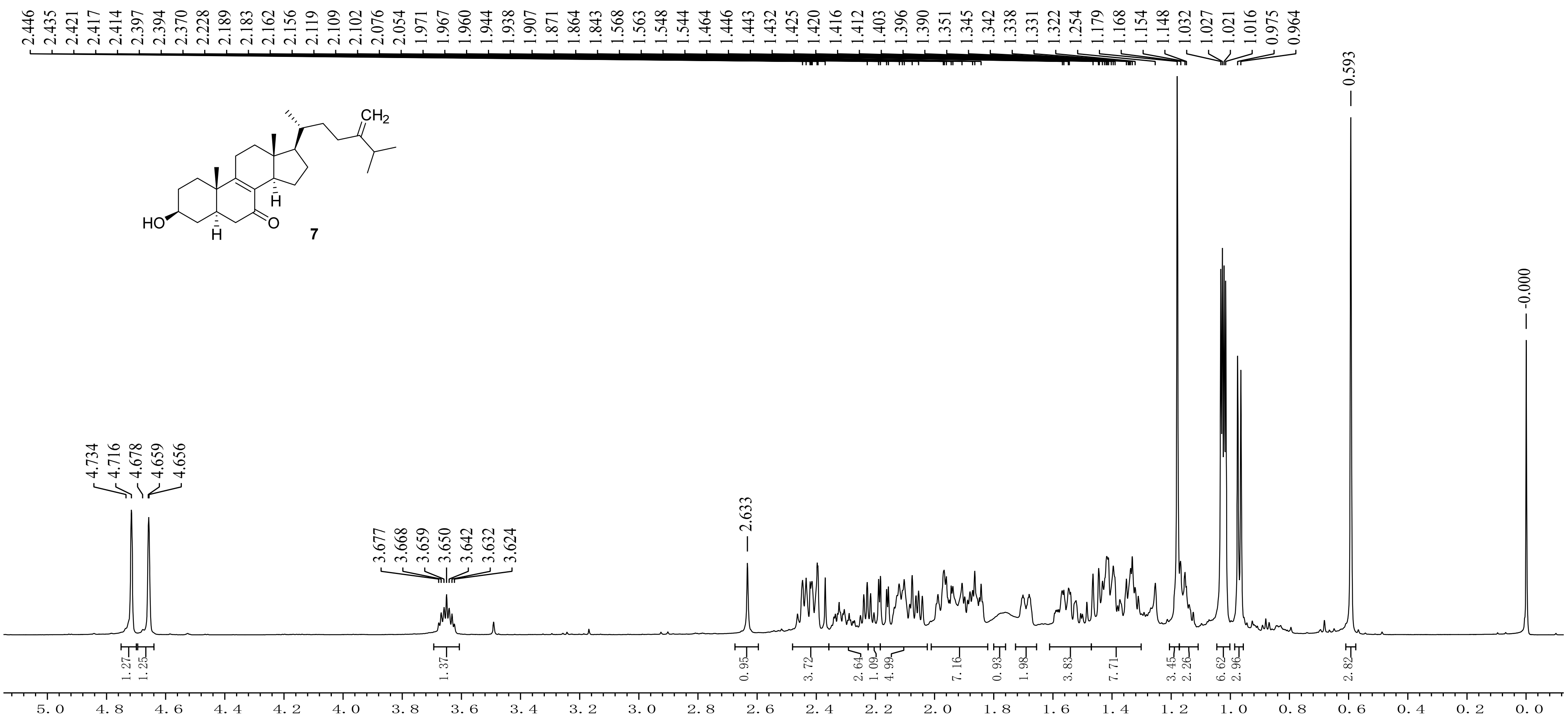

Figure S29. The ^{1}H NMR spectrum of 3β-hydroxyergosta-8,24(28)-dien-7-one (**7**) in $CDCl_3$ (600 MHz).

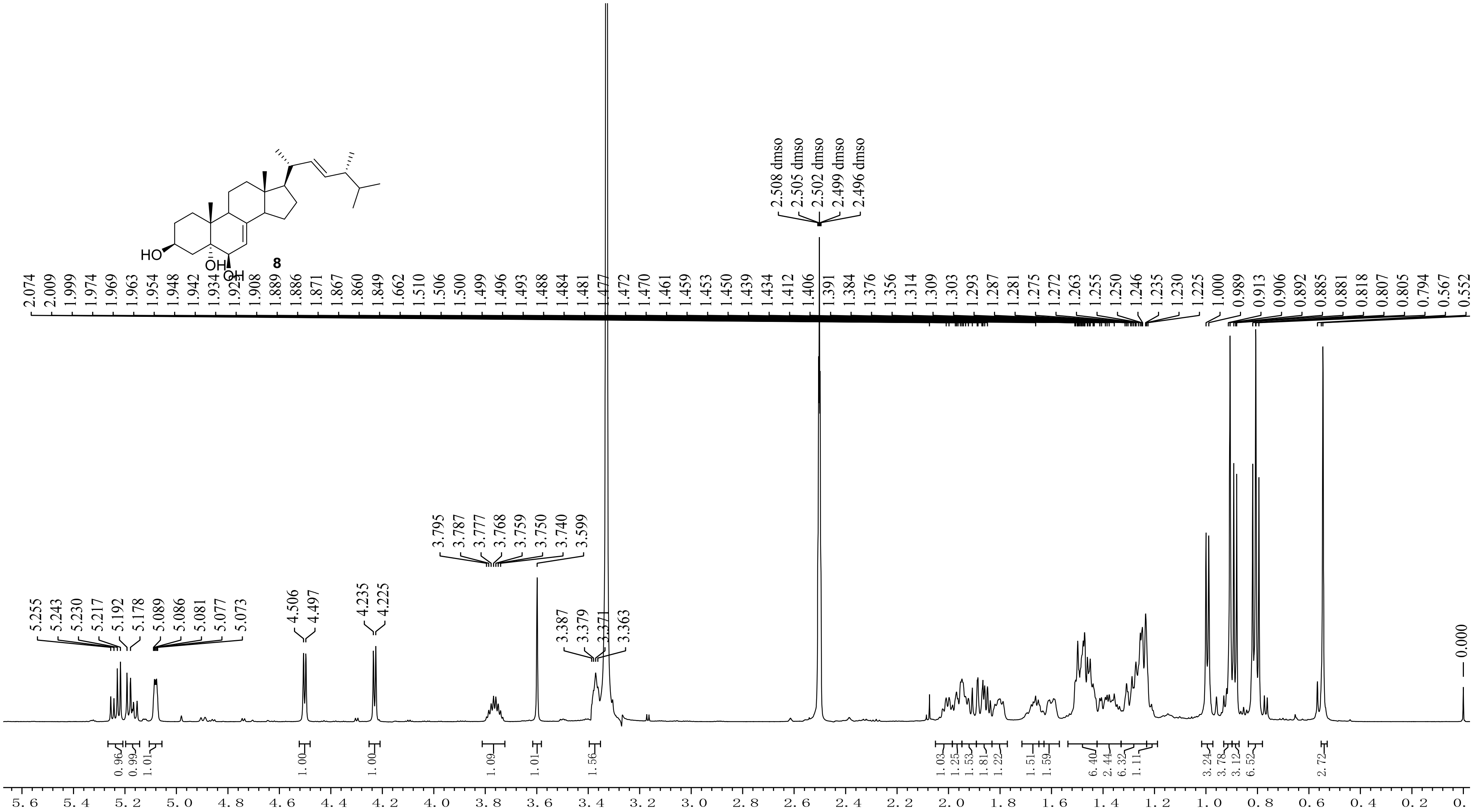

Figure S30. The ^{1}H NMR spectrum of (22*E*,24*R*)-24-methyl-5α-cholesta-7,22-dien-3*β*,5,6*β*-triol (**8**) in DMSO-d_6 (600 MHz).

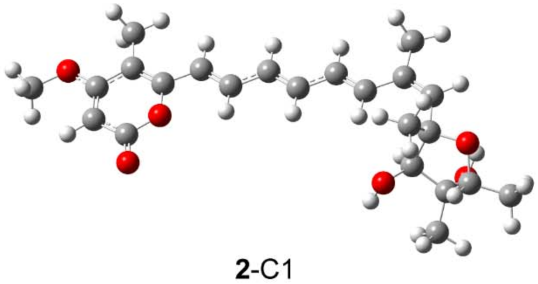

2-C1

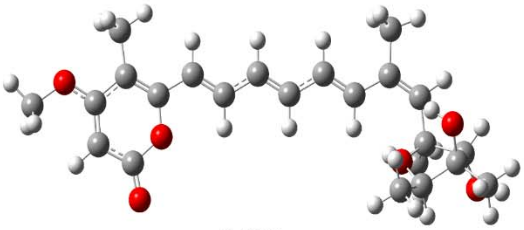

2-C2

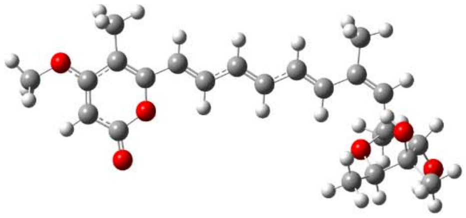

2-C3

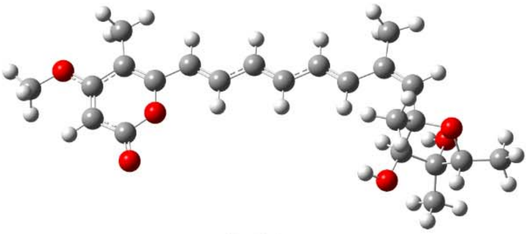

2-C4

Figure S31. Optimized conformers (≥ 1%) of 14*S*,15*R*,16*R*,17*R*-**2** at the M06-2X/6-311+G(d,p) level in MeOH

Table S2. Boltzmann populations of the identified conformers for 14*S*,15*R*,16*R*,17*R*-**2**

conformer	G (Hartree)	ΔG (Kcal/mol)	P (%)
**2-C1**	-1345.200346	0.0000	70.68
**2-C2**	-1345.199113	0.7737	19.13
**2-C3**	-1345.198076	1.4244	6.37
**2-C4**	-1345.197594	1.7269	3.82

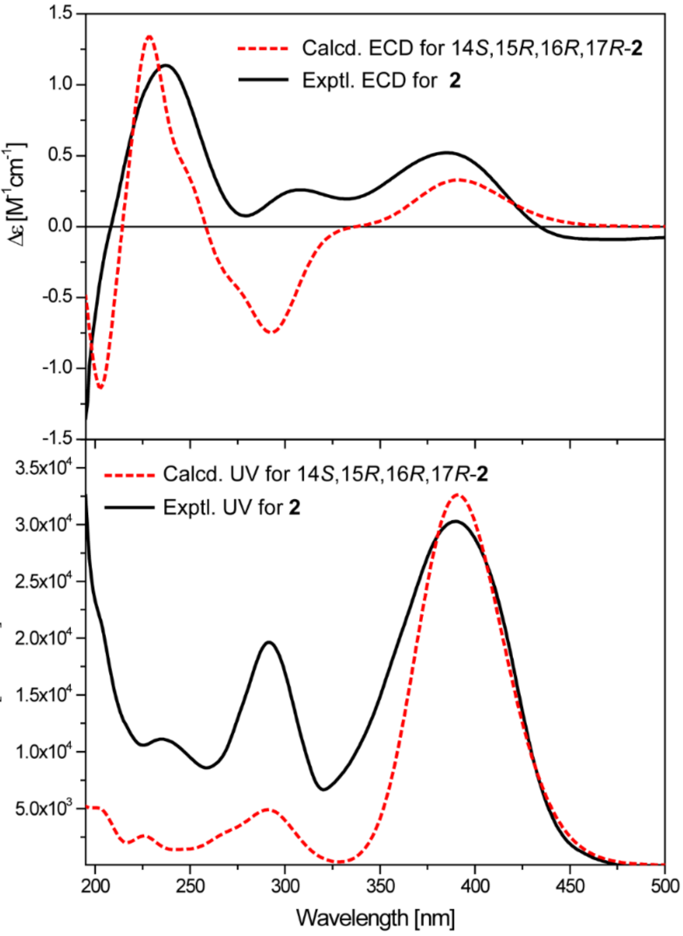

Figure S32. Comparison of the experimental and calculated ECD and UV spectra of **2**

Table S3. Calculated ECD data for conformers **2-C1** and **2-C2** at the Cam-B3LYP/TZVP//M06-2X/6-311+G(d,p) level in MeOH

Excited State	**2**-C1					**2**-C2				
	ΔE^a(eV)	λ^bnm	f^c	*Rlen*^{d}	*Rvel*^{e}	ΔE^a(eV)	λ^bnm	f^c	*Rlen*^{d}	*Rvel*^{e}
1	3.2976	376.2437	26.59694	30.6822	26.59694	3.3007	375.8903	26.62194	-27.7202	-27.0569
2	4.4601	278.1779	35.97314	-49.3939	35.97314	4.4844	276.67048	36.16913	41.9392	50.0977
3	4.8915	253.6443	39.45261	-17.6266	39.45261	4.8937	253.53027	39.47036	12.7071	15.5789
4	5.3433	232.1975	43.09662	17.3659	43.09662	5.4464	227.80205	43.92818	-12.2595	-14.8884
5	5.5655	222.9272	44.88879	8.0492	44.88879	5.454	227.48462	43.98948	-4.4356	-7.8779
6	5.5918	221.8787	45.10091	0.0352	45.10091	5.5976	221.64876	45.14769	-0.6408	-18.3102
7	5.791	214.2464	46.70757	45.2304	46.70757	5.5981	221.62896	45.15173	-20.9574	-0.2514
8	5.9024	210.2028	47.60607	10.4811	47.60607	5.8876	210.73121	47.4867	-17.5602	-11.223
9	6.0844	203.9151	49.074	7.6707	49.074	6.0524	204.99324	48.8159	-4.7534	-14.7205
10	6.3729	194.6839	51.40091	-6.5499	51.40091	6.3824	194.39413	51.47753	4.7904	6.2458
11	6.5552	189.2698	52.87126	-8.4791	52.87126	6.5325	189.92746	52.68817	2.4564	2.7974
12	6.6074	187.7745	53.29228	-15.1166	53.29228	6.6145	187.57292	53.34955	29.6829	21.8014
13	6.6288	187.1683	53.46488	-25.0253	53.46488	6.6199	187.41992	53.3931	5.7702	13.643
14	6.7281	184.4059	54.26579	4.362	54.26579	6.6733	185.92017	53.8238	-7.6567	-7.2471
15	6.9426	178.7084	55.99585	-12.9955	55.99585	6.9821	177.69741	56.31444	19.7644	13.9829
16	7.0426	176.1709	56.80241	2.2762	56.80241	7.0098	176.99522	56.53786	0.0296	12.2931
17	7.0559	175.8388	56.90968	-7.4568	56.90968	7.0461	176.08338	56.83063	2.1474	-3.776
18	7.1309	173.9894	57.51459	5.6598	57.51459	7.1247	174.14082	57.46459	-11.4836	5.9067
19	7.1685	173.0768	57.81786	11.7441	57.81786	7.1611	173.25566	57.75817	-5.9312	-24.3423
20	7.1836	172.713	57.93965	-0.8164	57.93965	7.2255	171.71145	58.27759	3.4868	10.7131
21	7.2896	170.2015	58.79459	4.2197	58.79459	7.2876	170.24824	58.77846	-4.8904	-15.3328
22	7.3856	167.9892	59.56889	11.5937	59.56889	7.3	169.95905	58.87848	-23.1811	-10.9228
23	7.4431	166.6915	60.03266	-0.6016	60.03266	7.4545	166.43653	60.1246	-0.1718	3.0013
24	7.5101	165.2043	60.57305	6.7348	60.57305	7.4853	165.75169	60.37302	-0.549	-5.2476
25	7.5651	164.0033	61.01665	-9.3323	61.01665	7.4994	165.44005	60.48675	4.1098	-0.8876
26	7.6214	162.7918	61.47074	-1.8717	61.47074	7.584	163.59455	61.16909	-4.679	-1.7144
27	7.6558	162.0603	61.7482	3.7813	61.7482	7.6259	162.69569	61.50704	-4.0529	-6.9897
28	7.6929	161.2787	62.04743	-0.8754	62.04743	7.6945	161.24519	62.06033	-3.3626	3.9216
29	7.7382	160.3346	62.4128	-5.3771	62.4128	7.7759	159.55723	62.71687	4.5799	-11.6033
30	7.8092	158.8769	62.98545	6.6686	62.98545	7.8701	157.64744	63.47664	1.0708	-6.2282
31	7.8506	158.039	63.31937	0.2737	63.31937	7.9016	157.01897	63.73071	1.7434	0.5937
32	7.9048	156.9554	63.75652	0.7906	63.75652	7.9332	156.39352	63.98558	4.092	5.4792
33	7.9968	155.1497	64.49855	8.5604	64.49855	8.015	154.79739	64.64534	-6.9712	-8.1076
34	8.0413	154.2911	64.85746	5.9997	64.85746	8.0216	154.67003	64.69857	-2.993	33.9608
35	8.083	153.4951	65.1938	1.5752	65.1938	8.0474	154.17416	64.90666	7.1064	-15.328
36	8.1454	152.3192	65.69709	-0.077	65.69709	8.1563	152.11568	65.785	-4.1604	-11.8535
37	8.2111	151.1005	66.22699	6.592	66.22699	8.1712	151.8383	65.90518	-11.0796	0.2826
38	8.2334	150.6912	66.40686	10.9372	66.40686	8.1764	151.74173	65.94712	-8.5864	-3.9928
39	8.2614	150.1805	66.63269	-9.6292	66.63269	8.2593	150.21867	66.61575	3.4222	5.3137
40	8.2883	149.6931	66.84965	-4.1519	66.84965	8.3126	149.25548	67.04565	4.8554	0.5139
41	8.3065	149.3651	66.99645	-5.7654	66.99645	8.3248	149.03675	67.14405	6.9202	5.8308
42	8.3358	148.8401	67.23277	-5.9394	67.23277	8.3419	148.73124	67.28197	-9.844	-2.4846
43	8.3977	147.743	67.73202	-9.433	67.73202	8.3727	148.18411	67.53039	-6.7282	-9.6101
44	8.4647	146.5736	68.27242	55.3182	68.27242	8.4203	147.34642	67.91431	-5.1754	1.297
45	8.4681	146.5147	68.29984	-43.1859	68.29984	8.4718	146.45071	68.32968	0.4926	-0.039
46	8.5163	145.6855	68.6886	8.7241	68.6886	8.5455	145.18765	68.92411	-2.0722	15.1272
47	8.5476	145.152	68.94105	-9.5148	68.94105	8.5891	144.45065	69.27577	-13.228	-18.5797
48	8.6067	144.1553	69.41772	7.6241	69.41772	8.613	144.04982	69.46854	7.7041	3.2253
49	8.6431	143.5482	69.71131	7.1232	69.71131	8.6318	143.73608	69.62017	11.3842	8.9334
50	8.6732	143.05	69.95408	-12.1333	69.95408	8.6514	143.41044	69.77825	2.1028	-5.5594
51	8.6825	142.8968	70.02909	16.6379	70.02909	8.6591	143.28292	69.84036	-14.1763	-12.8312
52	8.6901	142.7718	70.09039	-0.7292	70.09039	8.6891	142.78822	70.08232	1.4665	2.7739
53	8.7555	141.7053	70.61788	-10.9529	70.61788	8.7471	141.84142	70.55013	5.3073	6.5065
54	8.7764	141.3679	70.78645	5.5706	70.78645	8.756	141.69725	70.62191	2.8488	-2.8364
55	8.8444	140.281	71.3349	-26.1376	71.3349	8.8071	140.8751	71.03406	9.9859	-4.4145
56	8.8812	139.6997	71.63172	2.431	71.63172	8.8412	140.33175	71.30909	-26.6818	6.7013
57	8.9076	139.2857	71.84465	7.8742	71.84465	8.9028	139.36077	71.80593	-4.7461	-0.1488
58	8.9156	139.1607	71.90917	0.9461	71.90917	8.9078	139.28255	71.84626	-21.1992	-21.3012
59	8.9611	138.4541	72.27615	20.8241	72.27615	8.9295	138.94407	72.02128	-13.3779	26.5565
60	8.9745	138.2474	72.38423	5.0188	72.38423	8.9482	138.65371	72.17211	2.6145	-16.4936
61	8.9944	137.9415	72.54474	-0.1106	72.54474	8.9666	138.36918	72.32051	-4.0153	-6.8572
62	9.0275	137.4357	72.8117	-5.2499	72.8117	8.9909	137.99521	72.51651	4.2307	-5.5977
63	9.0634	136.8914	73.10126	0.8912	73.10126	9.0067	137.75313	72.64394	12.9026	3.2999
64	9.1071	136.2345	73.45372	10.3805	73.45372	9.0249	137.47533	72.79073	2.1937	-1.2043
65	9.128	135.9226	73.62229	-37.6371	73.62229	9.0878	136.52381	73.29806	-3.7226	-1.1551

[a] Excitation energy. [b] Wavelength. [c] Oscillator strength. [d] Rotatory strength in length form (10^{-40}cgs.). [e] Rotatory strength in velocity form (10^{-40}cgs.)

Table S4. Calculated ECD data for conformers **2-C3** and **2-C4** at the Cam-B3LYP/TZVP//M06-2X/6-311+G(d,p) level in MeOH

Excited State	**2**-C3					**2**-C4				
	ΔE^{a}(eV)	λ^{b}nm	*f*^{c}	*Rlen*^{d}	*Rvel*^{e}	ΔE^{a}(eV)	λ^{b}nm	*f*^{c}	*Rlen*^{d}	*Rvel*^{e}
1	3.302	375.74231	26.63243	-29.7383	-27.0569	3.2851	377.67529	26.49612	37.0784	35.56
2	4.4846	276.65814	36.17074	50.9863	50.0977	4.4416	279.33652	35.82392	-54.644	-54.1334
3	4.8918	253.62875	39.45503	14.4539	15.5789	4.9006	253.17331	39.52601	-17.6068	-19.0161
4	5.4268	228.62481	43.7701	-15.6514	-14.8884	5.3691	231.08176	43.30472	32.9852	31.8779
5	5.5074	225.27892	44.42018	-8.4063	-7.8779	5.5826	222.24431	45.02671	15.7506	15.8538
6	5.5903	221.9382	45.08881	-21.0214	-18.3102	5.5967	221.6844	45.14043	-0.4083	-0.8829
7	5.5961	221.70817	45.13559	0.2135	-0.2514	5.6892	218.08006	45.8865	16.3226	18.671
8	5.8684	211.42068	47.33184	-11.2003	-11.223	5.8967	210.40601	47.5601	26.4101	25.1807
9	5.9621	208.098	48.08758	-15.4801	-14.7205	6.3251	196.15518	51.01538	-1.0448	-0.902
10	6.3825	194.39108	51.47834	5.7689	6.2458	6.3792	194.49164	51.45172	-18.2743	-18.4525
11	6.5424	189.64006	52.76802	2.6323	2.7974	6.5595	189.14568	52.90594	-13.9813	-13.2417
12	6.6138	187.59278	53.3439	22.8562	21.8014	6.6143	187.57859	53.34793	13.3495	12.7452
13	6.6207	187.39727	53.39955	13.6934	13.643	6.6312	187.10054	53.48424	-31.4815	-31.111
14	6.7756	183.1131	54.6489	-7.5585	-7.2471	6.6791	185.75872	53.87058	-1.4983	-1.8785
15	6.9752	177.87319	56.25879	15.2235	13.9829	6.8559	180.96838	55.29657	49.4301	46.5908
16	7.0464	176.07588	56.83305	11.2333	12.2931	6.9602	178.25653	56.1378	-34.4539	-32.7823
17	7.0512	175.95602	56.87177	-3.4581	-3.776	7.04	176.23595	56.78143	-10.6236	-11.3604
18	7.1585	173.31859	57.7372	6.1476	5.9067	7.0735	175.4013	57.05163	-8.2446	-8.1184
19	7.1997	172.32678	58.0695	-25.6415	-24.3423	7.1745	172.93206	57.86625	27.6644	29.0409
20	7.2151	171.95896	58.19371	13.0369	10.7131	7.1796	172.80922	57.90738	-19.4111	-13.6695
21	7.2864	170.27628	58.76878	-14.1479	-15.3328	7.2205	171.83036	58.23727	2.4447	2.7343
22	7.3167	169.57113	59.01317	-10.9089	-10.9228	7.3252	169.37437	59.08173	-5.3183	-8.2406
23	7.4312	166.95838	59.93668	2.8303	3.0013	7.3675	168.40191	59.4229	-8.8837	-9.6797
24	7.467	166.15791	60.22542	-5.3312	-5.2476	7.3845	168.01423	59.56001	27.9468	27.1198
25	7.5034	165.35185	60.51901	-0.8652	-0.8876	7.4816	165.83366	60.34318	4.4116	3.7669
26	7.5932	163.39634	61.24329	-0.8576	-1.7144	7.5141	165.11639	60.60531	-13.3887	-14.1177
27	7.6319	162.56779	61.55543	-7.1353	-6.9897	7.5783	163.7176	61.12312	1.5571	1.6625
28	7.6763	161.62749	61.91354	3.8455	3.9216	7.6543	162.09204	61.7361	4.866	3.9092
29	7.7778	159.51826	62.73219	-10.7368	-11.6033	7.7759	159.55723	62.71687	-1.514	-1.4869
30	7.8546	157.95853	63.35163	-6.0094	-6.2282	7.8894	157.26178	63.63231	0.3567	0.3971
31	7.8893	157.26377	63.6315	0.5897	0.5937	7.96	155.86697	64.20174	36.619	36.8256
32	7.9965	155.15552	64.49613	1.6383	5.4792	7.9714	155.64407	64.29368	-2.8068	1.4381
33	8.0165	154.76843	64.65744	-7.6333	-8.1076	8.0209	154.68353	64.69293	-11.3348	-10.8863
34	8.0431	154.25658	64.87198	34.9041	33.9608	8.0612	153.91022	65.01797	12.5803	2.3388
35	8.0829	153.49702	65.19299	-17.9097	-15.328	8.0785	153.58063	65.1575	-11.7899	-8.7906
36	8.1555	152.1306	65.77855	-13.5585	-11.8535	8.1302	152.60401	65.57449	7.0535	7.8874
37	8.1575	152.0933	65.79468	0.4093	0.2826	8.1791	151.69164	65.9689	0.5682	0.8507
38	8.2095	151.12992	66.21409	-4.3734	-3.9928	8.2249	150.84695	66.3383	-21.055	-21.9605
39	8.2611	150.18594	66.63027	5.1336	5.3137	8.3009	149.46585	66.95128	0.125	-2.8764
40	8.2943	149.58479	66.89805	2.217	0.5139	8.3152	149.20881	67.06662	-19.0022	-16.5554
41	8.3077	149.34351	67.00613	7.8839	5.8308	8.3296	148.95086	67.18276	-8.9834	-8.3244
42	8.3425	148.72054	67.28681	-3.5951	-2.4846	8.3699	148.23368	67.5078	9.5459	7.3503
43	8.3921	147.84155	67.68686	-9.4444	-9.6101	8.3888	147.89971	67.66024	33.5765	34.3846
44	8.4447	146.92068	68.11111	3.0937	1.297	8.4791	146.32462	68.38856	-36.21	-37.1556
45	8.4561	146.72261	68.20305	6.1784	-0.039	8.4853	146.21771	68.43857	7.2811	6.6992
46	8.47	146.48183	68.31516	11.6749	15.1272	8.5166	145.68033	68.69102	11.3619	10.7885
47	8.4916	146.10923	68.48938	-17.3593	-18.5797	8.5388	145.30158	68.87007	3.2249	2.9824
48	8.5496	145.11803	68.95718	2.5	3.2253	8.5566	144.99931	69.01364	41.935	42.7775
49	8.5899	144.4372	69.28222	8.4729	8.9334	8.5602	144.93833	69.04268	-21.0096	-19.7734
50	8.6247	143.85441	69.5629	-6.2229	-5.5594	8.611	144.08328	69.45241	8.8159	8.6538
51	8.6462	143.49669	69.73631	-11.8613	-12.8312	8.6473	143.47844	69.74518	1.1613	1.4941
52	8.6706	143.09288	69.93311	2.7658	2.7739	8.6539	143.36901	69.79842	4.2839	4.463
53	8.6805	142.92968	70.01296	6.0681	6.5065	8.6799	142.93956	70.00812	4.9275	8.4591
54	8.7097	142.4505	70.24847	-2.9	-2.8364	8.7162	142.34427	70.3009	3.0867	6.8111
55	8.7528	141.74905	70.5961	-3.7776	-4.4145	8.7531	141.74419	70.59852	-3.7131	-4.8499
56	8.8109	140.81434	71.06471	7.5726	6.7013	8.792	141.11705	70.91227	7.6084	7.203
57	8.8595	140.04189	71.45669	-0.8242	-0.1488	8.8118	140.79996	71.07197	-4.0224	-3.3027
58	8.8755	139.78943	71.58574	-24.0941	-21.3012	8.8865	139.6164	71.67446	2.4636	2.408
59	8.909	139.26379	71.85594	28.9846	26.5565	8.9032	139.35451	71.80916	-0.5752	-2.6212
60	8.9203	139.08737	71.94708	-22.9018	-16.4936	8.9136	139.19192	71.89304	-8.0571	-5.662
61	8.9637	138.41395	72.29712	-10.2558	-6.8572	8.9351	138.85699	72.06645	-24.6495	-20.2925
62	8.9737	138.2597	72.37778	-1.9975	-5.5977	8.9452	138.70021	72.14791	-37.9874	-39.8575
63	8.9866	138.06124	72.48182	6.4346	3.2999	8.9612	138.45256	72.27696	-0.3881	-2.5053
64	9.0341	137.33533	72.86494	-0.8125	-1.2043	8.9798	138.16578	72.42698	-2.2297	-1.2313
65	9.0759	136.70282	73.20208	-0.3045	-1.1551	8.9923	137.97372	72.5278	11.337	9.381

^{a} Excitation energy. ^{b} Wavelength. ^{c} Oscillator strength. ^{d} Rotatory strength in length form (10^{-40}cgs.). ^{e} Rotatory strength in velocity form (10^{-40}cgs.)

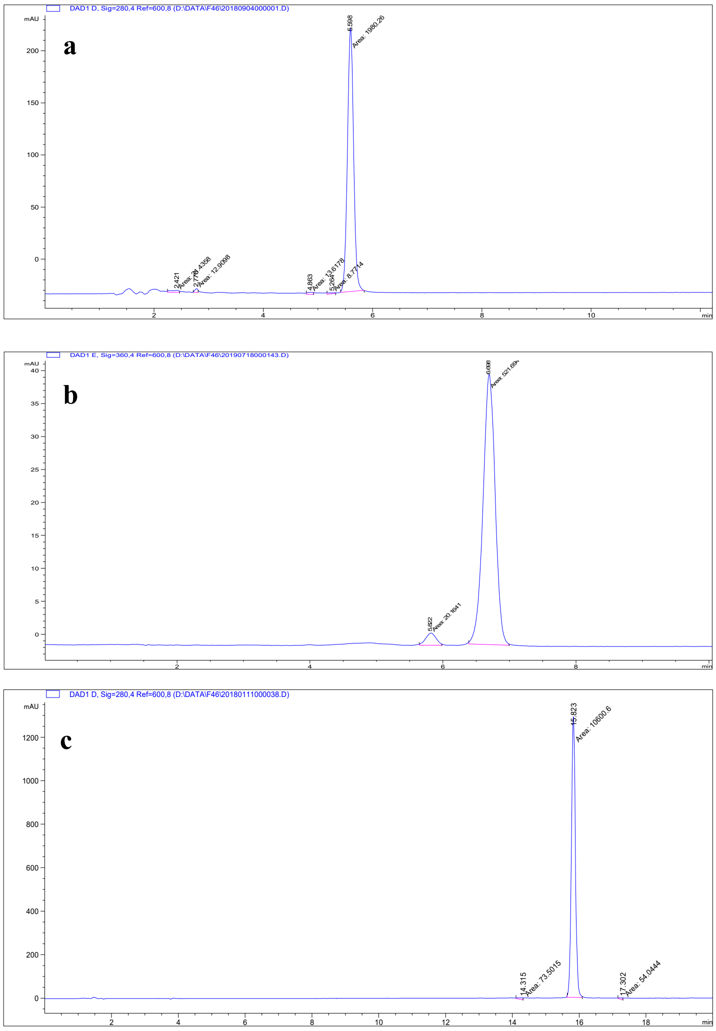

Figure S33. The HPLC analysis of **1–3** (**a**) Compound **1** (Capcell MGII 5μM, 3.0 mm × 150 mm, 65%ACN in 5 mM NH_4AC). (b) Compound **2** (Capcell MGII 5μM, 3.0 mm × 150 mm, 70%ACN in 5 mM NH_4AC). (**c**) Compound **3** (Capcell MGII 5μM, 3.0 mm × 150 mm, 0-10 min: 50-95%ACN in 5 mM NH_4AC; 10-20 min: 95%ACN in 5 mM NH_4AC 10min)
